# Androgen Insensitivity Syndrome with Bilateral Gonadal Sertoli Cell Lesions, Sertoli–Leydig Cell Tumor, and Paratesticular Leiomyoma: A Case Report and First Systematic Literature Review

**DOI:** 10.3390/jcm13040929

**Published:** 2024-02-06

**Authors:** Apollon I. Karseladze, Aleksandra V. Asaturova, Irina A. Kiseleva, Alina S. Badlaeva, Anna V. Tregubova, Andrew R. Zaretsky, Elena V. Uvarova, Magda Zanelli, Andrea Palicelli

**Affiliations:** 1 Oncopathology Department, National Medical Research Center for Obstetrics, Gynecology and Perinatology named after Academician V.I. Kulakov of the Ministry of Health of Russia, Bldg. 4, Oparina Street, Moscow 117513, Russia; 2 1st Pathology Department, National Medical Research Center for Obstetrics, Gynecology and Perinatology named after Academician V.I. Kulakov of the Ministry of Health of Russia, Bldg. 4, Oparina Street, Moscow 117513, Russia; 3 Pediatric Gynecology Department, National Medical Research Center for Obstetrics, Gynecology and Perinatology named after Academician V.I. Kulakov of the Ministry of Health of Russia, Bldg. 4, Oparina Street, Moscow 117513, Russia; 4 Department of Molecular Technologies, Research Institute of Translational Medicine, N. I. Pirogov Russian National Research Medical University of the Ministry of Health of the Russian Federation, Bldg. 1, Ostrovityanova Street, Moscow 117997, Russia; a-zaretsky@yandex.ru; 5 Pathology Unit, Azienda USL—IRCCS di Reggio Emilia, 42123 Reggio Emilia, Italy; magda.zanelli@ausl.re.it (M.Z.);

**Keywords:** androgen insensitivity syndrome (AIS), CAIS, testicular feminization, Sertoli cell adenoma, Sertoli cell hamartoma, Sertoli–Leydig cell tumor, leiomyoma, androgen receptor, persistence of fallopian tubes in CAIS

## Abstract

Androgen insensitivity syndrome (AIS) is a rare Mendelian disorder caused by mutations of the androgen receptor (*AR*) gene on the long arm of the X chromosome. As a result of the mutation, the receptor becomes resistant to androgens, and hence, karyotypically male patients (46,XY) carry a female phenotype. Their cryptorchid gonads are prone to the development of several types of tumors (germ cell, sex cord stromal, and others). Here, we report a 15-year-old female-looking patient with primary amenorrhea who underwent laparoscopic gonadectomy. Histologically, the patient’s gonads showed Sertoli cell hamartomas (SCHs) and adenomas (SCAs) with areas of Sertoli–Leydig cell tumors (SLCTs) and a left-sided paratesticular leiomyoma. Rudimentary Fallopian tubes were also present. The patient’s karyotype was 46,XY without any evidence of aberrations. Molecular genetic analysis of the left gonad revealed two likely germline mutations—a pathogenic frameshift deletion in the *AR* gene (c.77delT) and a likely pathogenic missense variant in the *RAC1* gene (p.A94V). Strikingly, no somatic mutations, fusions, or copy number variations were found. We also performed the first systematic literature review (PRISMA guidelines; screened databases: PubMed, Scopus, Web of Science; ended on 7 December 2023) of the reported cases of patients with AIS showing benign or malignant Sertoli cell lesions/tumors in their gonads (*n* = 225; age: 4–84, mean 32 years), including Sertoli cell hyperplasia (1%), Sertoli cell nodules (6%), SCHs (31%), SCAs (36%), Sertoli cell tumors (SCTs) (16%), and SLCTs (4%). The few cases (*n* = 14, 6%; six SCAs, four SCTs, two SLCTs, and two SCHs) with available follow-up (2–49, mean 17 months) showed no evidence of disease (13/14, 93%) or died of other causes (1/14, 7%) despite the histological diagnosis. Smooth muscle lesions/proliferations were identified in 19 (8%) cases (including clearly reported rudimentary uterine remnants, 3 cases; leiomyomas, 4 cases). Rudimentary Fallopian tube(s) were described in nine (4%) cases. Conclusion: AIS may be associated with sex cord/stromal tumors and, rarely, mesenchymal tumors such as leiomyomas. True malignant sex cord tumors can arise in these patients. Larger series with longer follow-ups are needed to estimate the exact prognostic relevance of tumor histology in AIS.

## 1. Introduction

Androgen insensitivity syndrome (AIS) is a rare disease caused by mutations of the androgen receptor (*AR*) gene located on the long arm of the X chromosome (Xq 11–12) [1,2,3,4]. In a study, the incidence of AIS was 1:99,000 in genetically confirmed males [4]. However, not all the reported studies included a molecular proof of the diagnosis, and there is a need for new clinic pathological data to elaborate proper conceptual approaches to this disease. 

Clinically, these patients are genotypically males with a 46,XY karyotype, but they phenotypically present as females lacking the Müllerian derivates. Indeed, female-looking breasts and vulva are usually normal, while body hair could be less pronounced. The gonads are usually cryptorchid, identified in abdomino-pelvic or inguinal sites, while the uterus is absent and the vagina is typically blind and shortened; about 10% of patients may have one or both residual or well-formed Fallopian tubes [2]. 

Due to various types of mutations occurring in the locus of the *AR* gene (insertions and deletions), the phenotypical manifestations of the AIS may differ substantially; so, the disease itself is divided into broad entities, including complete (CAIS) and partial (PAIS) forms [3]. Moreover, the clinical manifestations depend on the patient’s age. In infants and pre-menarchal cases, the disease may be suspected during casual ultrasound investigations of the pelvis carried out for different reasons or manifested by inguinal hernias, including undescended testes. In menarchal patients, primary amenorrhea is the basic manifestation of the process. 

Finally, single or multiple hamartomas or tumors may rarely develop in the cryptorchid gonads, including malignant neoplasms, which can present as pelvic masses or even cause distant metastases. In particular, various lesions containing Sertoli and/or Leydig cells have been described in patients with AIS, considered as hyperplastic, hamartomatous, or neoplastic, including Sertoli cell hyperplasia (SCHYP), Sertoli cell nodules (SCNs), Sertoli cell hamartomas (SCHs), Sertoli cell adenomas (SCAs), Sertoli cell tumors not otherwise specified (SCTs, NOS) or Sertoli–Leydig cell tumors (SLCTs); single or multiple, uni or bilateral, these lesions may appear as grossly detectable nodules or incidental histological findings. However, their diagnostic criteria may be subtle, challenging, and sometimes questionable in some cases, and their frequently small size and bland histology may result in a favorable prognosis; unfortunately, the rarity of these lesions and the lack of a systematic literature review on this topic have represented biases for further considerations [5,6,7].

We here present an unusual case of bilateral gonadal SCHs with associated SCAs, SLCT, and paragonadal leiomyoma in a patient with molecular confirmation of CAIS. Moreover, we performed the first systematic literature review of the features of Sertoli cell lesions in patients with AIS.

## 2. Case Description

A 15-year-old female-looking patient was referred to the gynecologist due to primary amenorrhea. Her maternal grandaunt had uterine agenesis. The patient was born after unremarkable gestation and delivery (unattended childbirth, timely delivered). At the age of 2 years, she underwent surgery for umbilical and left inguinal hernias; no nodules were found in the hernia sacs. At presentation, the patient did not have any evidence of chronic diseases and did not take any drugs on a regular basis; previous drug history was negative as well.

Upon clinical examination, the patient had a typical feminine habitus, showing well-developed breasts with pale nipples. Pubic hair was absent. A 6 cm long vaginal stump with a blind end was found during the gynecological exam. During the bimanual rectal–abdominal investigation, the uterine body was not detected in the pelvis. Bilateral nodules (presumable gonads)—each of 4 cm in maximum size—were palpable at a high level of the pelvis, almost reaching the pelvic brim. Any other remarkable finding was identified on clinical examination, except for myopia of medium level.

**Figure 1:**
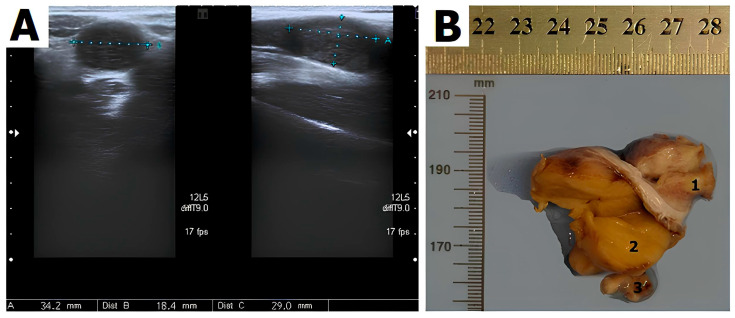
(**A**) Transvaginal ultrasonography demonstrating the gonads (testes) located medial to iliac vessels. The right gonad measured 31 × 12 × 11 mm, the left gonad 34 × 18 × 21 with medium-level echogenicity. (**B**) Gross examination of the left gonad: leiomyoma (1), Sertoli–Leydig tumor (2), fallopian tube (3) (previously unpublished original photos).

Ultrasonographic investigation confirmed the uterine agenesis and identified the gonads at the entrance of the pelvis. The right gonad measured 31 × 12 × 11 mm, while the left one measured 34 × 18 × 21 mm, showing a nodule measuring 18 × 14 mm at one of the poles (Figure 1).

The serum testosterone level (15 nmol/L) exceeded the typical normal values for female patients (2.3 nmol/L), but it corresponded to the normal rates for boys of the same age. The serum levels of Luteinizing hormone (LH) (37.2 IU/L; normal values: 2.4–8.3 IU/L) and anti-Müllerian hormone (AMH) (600 ng/mL; normal values: 10.6 ng/mL) were increased.

The patient underwent laparoscopic bilateral gonadectomy. During the procedure, the undescended gonads were found at the pelvic sidewalls.

Grossly, the right and left gonads had lobulated cut sections and measured 3.5 × 2.0 × 2.0 cm and 4.0 × 4.0 × 3.0 cm, respectively; the nodule adjacent to the left gonad measured 1.5 × 1.0 × 1.0 cm (Figure 1B). The routinely processed tissue blocks were formalin-fixed and paraffin-embedded.

**Figure 2:**
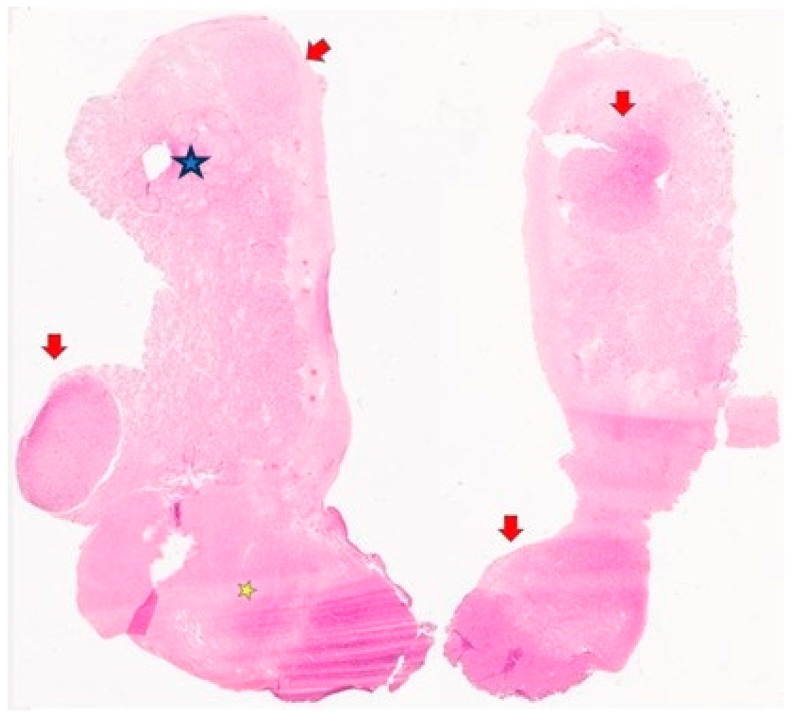
Histological examination of the right (on the right) and left (on the left) gonads: multinodular appearance (red arrows); Sertoli–Leydig tumor (blue star), leiomyoma (yellow star) (hematoxylin and eosin, macrosection) (previously unpublished original photos).

Upon histological examination, both gonads revealed multiple nodules arising in a hamartomatous background (Figure 2).

**Figure 3:**
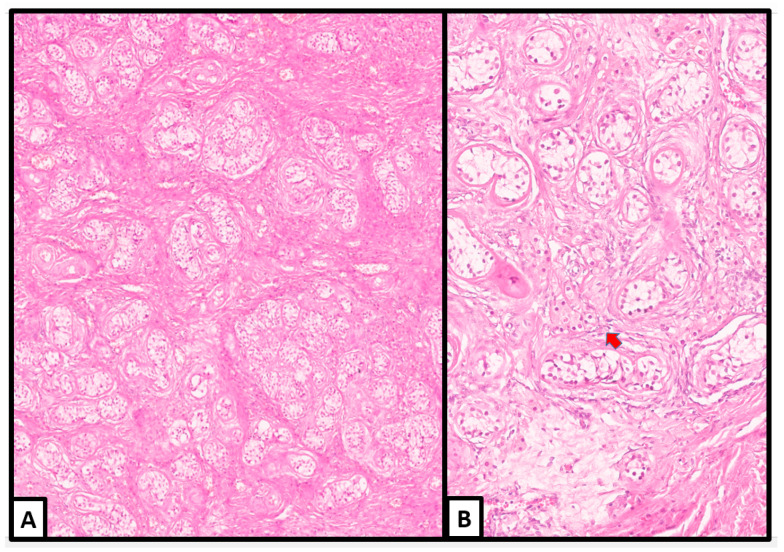
Histological examination. Hamartomatous sex cord structures containing Sertoli cell tubules in a fibromatous stroma. Clusters of Leydig cells are detected (red arrow) (hematoxylin and eosin; (**A**): 10×; (**B**): 20×) (previously unpublished original photos).

Microscopically, the hamartomatous background was composed of an extensive proliferation of Sertoli, Leydig, and stromal cells, which were identified in variable proportions in different areas and surrounded by a loose or fibromatous stroma (Figure 3); in some areas, ovarian-type stroma was also identified.

The Sertoli cells were arranged in compressed tubules separated by fibrous bands with foci of hyalinization and edema. Many tubules were wrapped by concentric layers of fibroblasts.

Leydig cells were dispersed throughout the tubules in varying proportions. They were polygonal in shape, showing sharp cellular borders, broad granular light-brown cytoplasm, and centrally located round nuclei with inconspicuous nucleoli. We detected Reinke crystalloid in a small number of Leydig cells.

Spermatogonia were not found in any tubules of the gonads, in spite of careful search.

**Figure 4:**
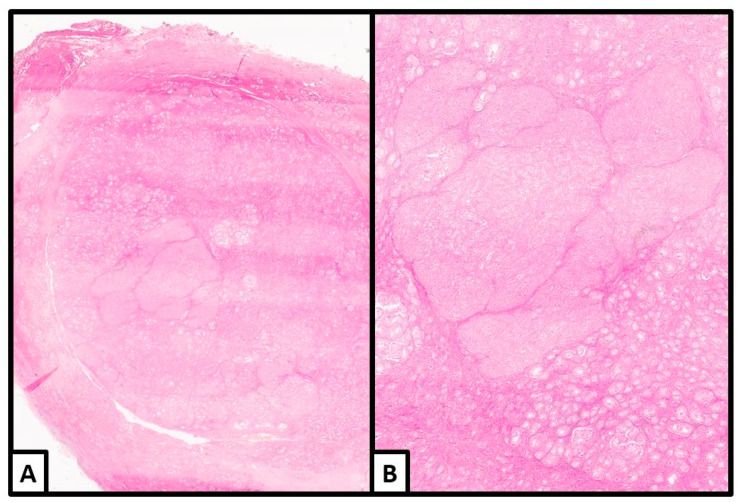
Histological examination. Unencapsulated, well-delimited nodule with areas of solid/complex arrangement of Sertoli cells (hematoxylin and eosin; (**A**): 2×; (**B**): 4×) (previously unpublished original photos).

**Figure 5:**
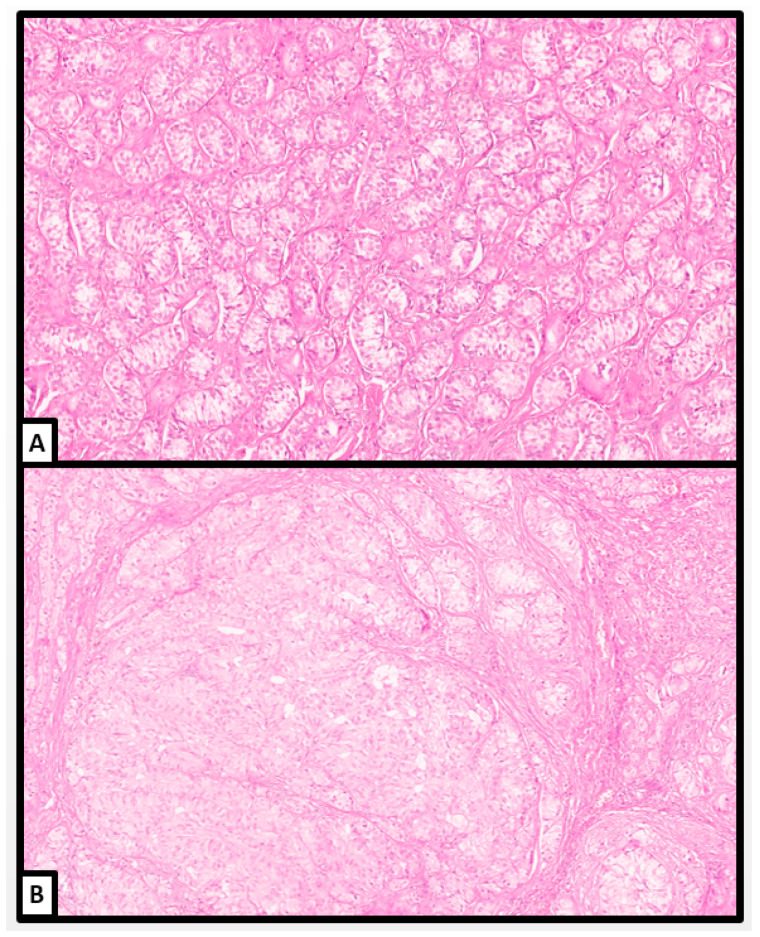
Histological examination. Details of other nodules showing a diffuse or more confluent growth of Sertoli cells with less evident Leydig cells. Some lumens were identified within a more complex growth (**B**) (hematoxylin and eosin; (**A**): 10×; (**B**): 10×) (previously unpublished original photos).

The transition from the hamartomatous background to the tumoral nodules manifested with significant enlargement of the Sertoli cells, which often acquired a round acinar-like, complex/confluent, or solid pattern (Figure 4 and Figure 5).

**Figure 6:**
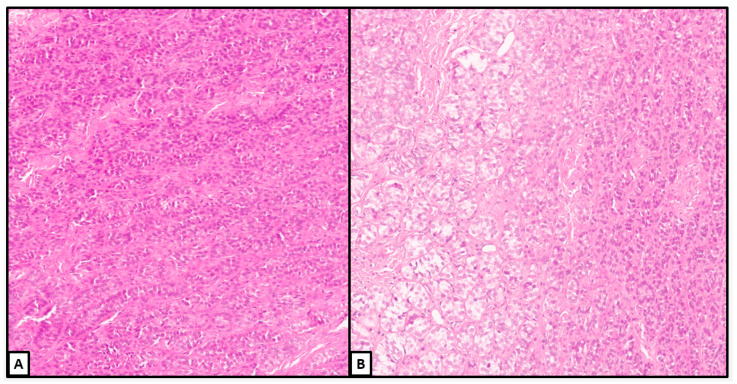
Histological examination of the sex cord–stromal tumor. (**A**) Cellular proliferation of solid tubules and cords of Sertoli cells in a fibromatous stroma. (**B**) Some cells had a clear cytoplasm (left) (hematoxylin and eosin; (**A**): 10×; (**B**): 10×) (previously unpublished original photos).

**Figure 7:**
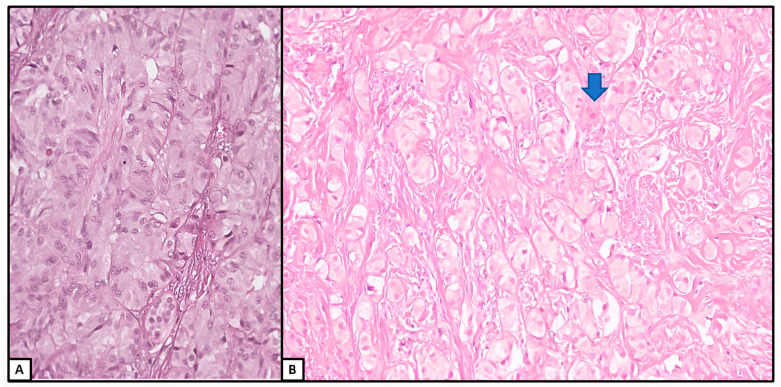
Histological examination. (**A**,**B**) Details of the Sertoli cells with eosinophilic cytoplasm. Rare Leydig cells with pink cytoplasm seemed to be intermixed (blue arrow) (hematoxylin and eosin; (**A**): 30×; (**B**): 20×) (previously unpublished original photos).

All the nodules were unencapsulated. Most of them were well circumscribed, while one of the nodules in the left gonad seemed to show a more cellular and complex/diffuse pattern of growth, worth a diagnosis of a sex cord–stromal tumor; in our opinion, we favored a well-differentiated SLCT (Figure 6 and Figure 7).

The Sertoli cells varied in size from cuboid to high prismatic, usually arranged in cords and solid tubules; depending on the areas, their cytoplasm was slightly eosinophilic, dense, or with signs of gradual vacuolization starting from apical to basal compartments. We also encountered some lipid-rich Sertoli cells. The nuclear/cytoplasmic ratio of tumor cells was very low. The nuclei were basally or centrally located, round in shape, with pale, finely dispersed chromatin and small, sometimes eosinophilic nucleoli. Some Leydig cells seemed to be intermixed.

The mitotic activity was still low in any areas of the sex cord–stromal tumor, as well as in the well-delimited nodules and in the hamartomatous background, revealing a maximum of 2 mitoses per 10 high-power field (HPF). Necrosis or other signs of discirculatory events were absent in any areas.

Immunohistochemical staining was performed on a Ventana BenchMark XT stainer using the following antibodies: alpha-fetoprotein (AFP) (Cell Marque, Rocklin, CA, USA), caldesmon (clone E-89, Cell Marque, Rocklin, CA, USA), calretinin (clone SP65, Ventana Medical-Systems, Oro Valley, AZ, USA), CD 117/c-kit (clone YR 145, Cell Marque, Rocklin, CA, USA), desmin (clone DE-R-11, Ventana Medical-Systems, Oro Valley, AZ, USA), alpha inhibin (clone R1, Cell Marque, Rocklin, CA, USA), Ki-67 (clone 30-9, Ventana Medical-Systems, Oro Valley, AZ, USA), MART-1/melan-A (clone A103, Ventana Medical-Systems, Oro Valley, AZ, USA), Oct-4 (clone MRQ-10, Cell Marque, Rocklin, CA, USA), PLAP (clone NB10, Cell Marque, Rocklin, CA, USA), and smooth-muscle actin (clone 1A4, Cell Marque, Rocklin, CA, USA).

**Figure 8:**
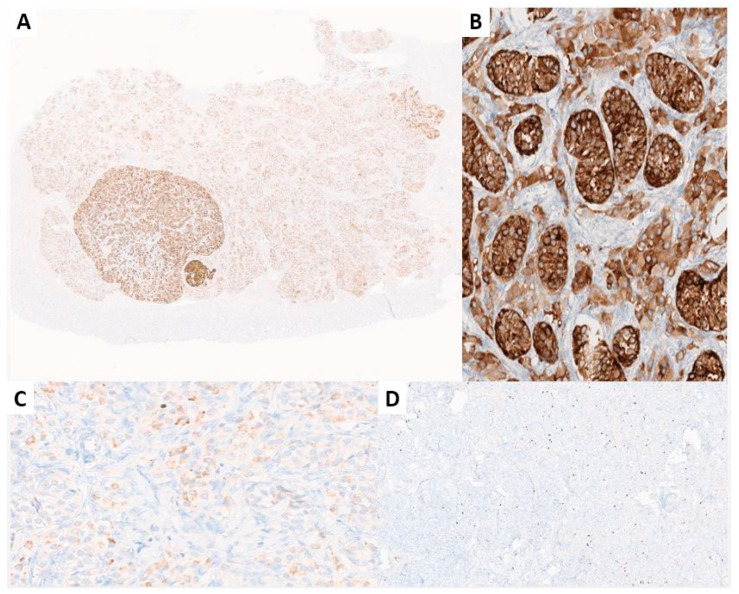
Immunohistochemical stainings. (**A**) Alpha inhibin was positive in the sex cord cells of the background hamartomatous component and tumor nodules (alpha inhibin, 2×); (**B**) detail of the hamartomatous background: alpha inhibin was positive in the hamartomatous Sertoli cell tubules and in intertubular Leydig cells (alpha inhibin, 20×). (**C**) Weak, patchy positivity for melan-A in the Sertoli cells (melan-A, 10×); (**D**) the proliferation index was low (<5%) in any area (Ki-67, 4×) (previously unpublished original photos).

Immunohistochemical stainings specific for germ cells (Oct-4, PLAP, c-kit) were negative in all the sex cord cells and did not reveal any in situ or invasive germ cell neoplasia. Conversely, marked positive reaction for inhibin-α, calretinin, and Melan-A was disclosed in Sertoli and Leydig cells; the proliferative index, according to the Ki67 level, was low, up to 4% mainly in basally located Sertoli cells (Figure 8).

**Figure 9:**
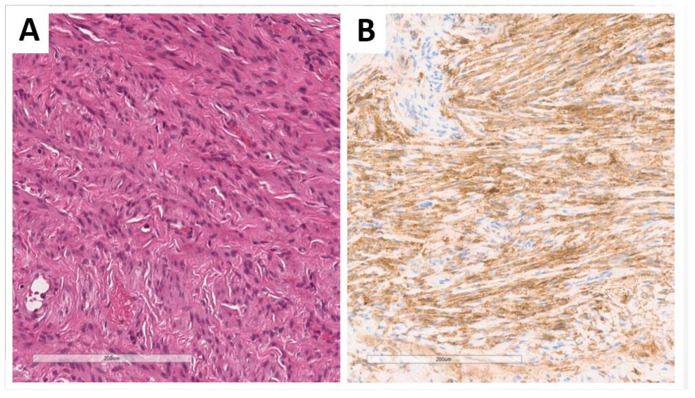
Histology of the paragonadal nodule, which was composed of fascicles of bland spindle cells (**A**) positive for smooth muscle markers (**B**) ((**A**): hematoxylin and eosin; (**A**): 10×; (**B**): immunohistochemistry for smooth muscle actin, 10×) (previously unpublished original photos).

The 1.5 cm paragonadal nodule (Figure 1, Figure 2 and Figure 9) was represented by bland, elongated cells arranged in fascicular structures and reminiscent of a smooth muscle neoplasm; mitotic activity and necrotic areas were not found.

These cells were positive to smooth muscle markers (desmin, smooth-muscle actin, caldesmon) (Figure 9) with a low proliferating index (Ki-67: 1%).

**Figure 10:**
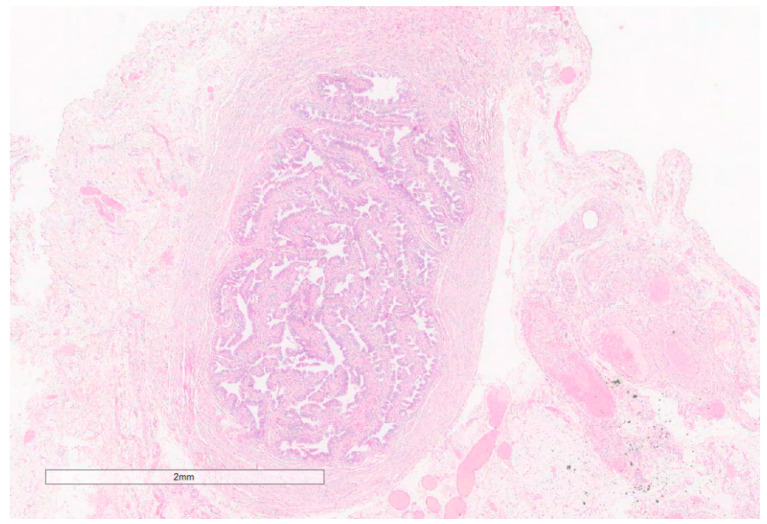
The rudimentary Fallopian tubes revealed a well-shaped wall of the uterine tube containing all three types of epithelial cells in the mucosa (Hematoxylin and eosin, 4×; previously unpublished, original photo).

Bilateral rudimentary fallopian tubes were also identified in the paragonadal tissues (Figure 10).

Cytogenetic analysis revealed a 46,XY karyotype.

Next-generation sequencing (NGS) analysis of the tumor tissue (Table 1) searched for DNA mutations of 523 genes and 127 microsatellite loci by using the NextSeq 550 instrument (Illumina, San Diego, CA, USA) with the TruSight Oncology 500 DNA+RNA reagent kit (Illumina, San Diego, CA, USA) in accordance with the instructions of the manufacturer.

NGS analysis identified a pathogenic variant in the *AR* gene (frameshift deletion generating a stop codon at position 34) and another likely pathogenic missense variant in the *RAC1* gene, while no mutations were detected in any of the other 521 genes tested; both variants represented likely germline mutations. Strikingly, no somatic mutations, translocations, or copy number variations were identified in the tumor sample. Pathogenic germline *AR* variant confirms the diagnosis of classic AIS.

## 3. Systematic Literature Review

### 3.1. Systematic Literature Review Method

**Figure 11:**
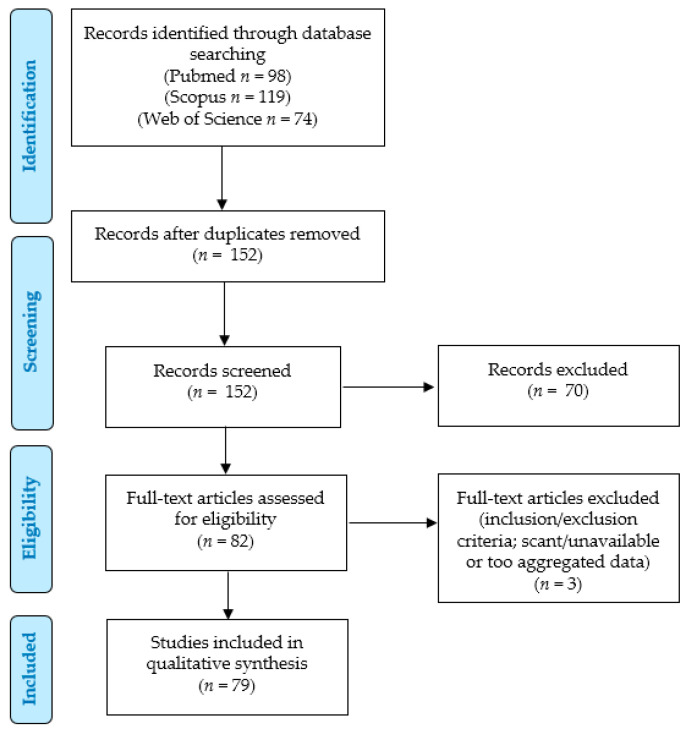
Systematic literature review: PRISMA flow chart.

We conducted a systematic literature review according to the PRISMA (“Preferred Reporting Items for Systematic Reviews and Meta-Analyses”) guidelines (http://www.prisma-statement.org/; accessed on 7 December 2023) to identify the previously reported cases of Sertoli cell lesions in patients with AIS (Figure 11).

Our retrospective observational study was conducted via the PICO process:

- Populations: human patients with AIS with a diagnosis of a gonadal Sertoli cell lesion;
- Intervention: any;
- Comparison: none;
- Outcomes: clinical outcomes (status at last follow-up, and survival and recurrence rates).

We searched for (“androgen insensitivity syndrome” OR “androgen resistance syndrome” OR “testicular feminization syndrome” OR “androgen receptor deficiency” OR “androgen insensitive syndrome” OR “Morris syndrome”) AND (Sertoli OR Sertoli-Leyding OR “sex-cord” OR “sex cord”) AND (tumor OR tumors OR tumour OR tumours OR nodule OR nodules OR adenoma OR adenomas OR hamartoma OR hamartomas) in the PubMed (all fields; 98 results; https://pubmed.ncbi.nlm.nih.gov, accessed on 7 December 2023), Scopus (Title/Abstract/Keywords; 119 results; https://www.scopus.com/home.uri, accessed on 7 December 2023) and Web of Science (Topic/Title; 74 results; https://login.webofknowledge.com, accessed on 7 December 2023) databases. No limitations or additional filters were set. The bibliographic research ended on 7 December 2023. We applied the following criteria:

- Eligibility/inclusion criteria: studies describing cases of patients with AIS with gonadal lesions containing Sertoli cells.
- Exclusion criteria: unclear tumor diagnosis; unclear AIS diagnosis; non-analyzable results (aggregated data); unavailable data (from full-text or abstracts).

Two independent authors removed the duplicates and checked the titles and abstracts of all the retrieved results (*n* = 152). After applying the eligibility, inclusion, and exclusion criteria, they selected 82 relevant eligible papers; 74 articles were retrieved in full-text format, and their reference lists were manually examined to check for other potentially relevant studies [1,6,7,8,9,10,11,12,13,14,15,16,17,18,19,20,21,22,23,24,25,26,27,28,29,30,31,32,33,34,35,36,37,38,39,40,41,42,43,44,45,46,47,48,49,50,51,52,53,54,55,56,57,58,59,60,61,62,63,64,65,66,67,68,69,70,71,72,73,74,75,76,77,78], while only abstracts or titles were available for the remaining 8 papers [79,80,81,82,83,84,85,86]. Five of these eight articles reported relevant data in their abstract and were included in further analysis [79,80,81,82,83], while three references were excluded due to insufficient data, according to the inclusion/exclusion criteria [84,85,86]. Finally, 79 articles were included in our study [1,6,7,8,9,10,11,12,13,14,15,16,17,18,19,20,21,22,23,24,25,26,27,28,29,30,31,32,33,34,35,36,37,38,39,40,41,42,43,44,45,46,47,48,49,50,51,52,53,54,55,56,57,58,59,60,61,62,63,64,65,66,67,68,69,70,71,72,73,74,75,76,77,78,79,80,81,82,83], although 8 papers retrieved in full-text format were just included for the analysis of a few parameters, as they reported partially aggregated data [6,72,73,74,75,76,77,78]. The extracted results were checked and confirmed by two other authors. Data collection was study- and case-related. Categorical variables were analyzed as frequencies and percentages, whereas continuous variables were by ranges and mean values. Meta-analysis was not performed according to the few available data for comparisons, especially concerning follow-up. This study was not recorded in PROSPERO (https://www.crd.york.ac.uk/prospero/, accessed on 27 January 2024).

### 3.2. Systematic Literature Review Results: Overview

Globally, 225 patients with AIS with lesions containing Sertoli cells have been reported according to our systematic literature review [1,6,7,8,9,10,11,12,13,14,15,16,17,18,19,20,21,22,23,24,25,26,27,28,29,30,31,32,33,34,35,36,37,38,39,40,41,42,43,44,45,46,47,48,49,50,51,52,53,54,55,56,57,58,59,60,61,62,63,64,65,66,67,68,69,70,71,72,73,74,75,76,77,78,79,80,81,82,83]. Most of the cases have been described in Europe (90/225, 40%) (32 United Kingdom [35,38,54,69,77], 19 United Kingdom or The Netherlands [26], 9 Spain [30,31,32,55,56], 7 France [78,80], 6 Poland [1,18,20,47,53,82], 6 Italy [43,46,62], 1 France or The Netherlands or Germany or Sweden [17], 1 Italy or France or Germany or Poland [29], 3 Czech Republic [12,79], 3 Germany [50,58], 1 Denmark [70], 1 Hungary [65], and 1 Ireland [63]), followed by North America (83/225, 37%) (82 United States [6,7,13,21,25,36,37,39,42,49,56,59,64,67,68,70,71,74] and 1 Canada [57]) and Asia (41/225, 18%) (22 China [10,11,22,72,73,75], 7 India [9,14,16,24,27,83], 3 Turkey [28,41,48], 2 Korea [45,51], 2 Japan [15,81], 2 Iran [19,32], 1 Russia, 1 Nepal [40], and 1 Taiwan [52]). To the best of our knowledge, we are here reporting the first Russian case in English literature. Only sporadic cases were described in Australia (7 cases) [44,61], Africa (1 Morocco [34], 1 Tunisia [46], 1 Nigeria [8]), and Brazil (1 case) [23].

Globally, 69 (31%) SCHs, 80 (36%) SCAs, 14 (6%) SCNs, 35 (16%) SCTs, 9 (4%) SLCTs, and 5 sex cord–stromal tumors containing some Sertoli cells (including 2 malignant cases) were reported and sometimes associated. However, most of them were case reports, while few studies reported the incidence of these lesions in patients with AIS. Moreover, these few series had some selection biases; indeed, they reported an 8.5% incidence of SCTs in patients with AIS (19/223 cases, range 5–15%, apparently more frequent in CAIS than PAIS individuals: 15/27, 12% vs. 2/55, 3%) [72,73,75], but SCTs are actually rarer in absolute number if compared to other lesions such as SCH or SCAs.

The patients’ ages ranged from 4 to 84 (mean 32) years. Only 6 patients were under 10 years of age (2 SCT, NOS [15,19]; 3 nodular and diffuse Sertoli cell lesions, possible SCAs? [55]; 1 SCN [61]); follow-up was available only for a 5-year-old patient who did not reveal evidence of disease 6 months after surgery [19].

Table 2 reports the results of the series with partially aggregated data, while Table 3 and Table 4 highlight the clinic-pathologic data of the cases described in detail.

Globally, our series included 156/225 (69%) CAIS [1,6,7,8,9,10,11,12,13,14,15,16,17,18,19,20,21,22,23,24,26,27,28,30,31,32,33,34,35,36,37,38,39,40,41,42,43,45,46,47,51,52,53,55,56,57,58,59,62,63,64,65,66,68,69,70,72,73,77,80,83], 9 (4%) PAIS [6,13,55,72,73], and 60 (27%) AIS, NOS patients [25,26,29,44,48,49,50,54,61,67,71,74,75,78,79,81,82]. Karyotype/fluorescent in situ hybridization analysis was performed in 58 CAIS [1,8,9,10,12,14,15,16,18,19,20,21,22,23,24,28,30,32,33,34,36,37,38,39,40,41,42,43,45,46,47,51,52,53,55,56,57,59,62,63,64,65,66,68,69,70,80,83], 1 PAIS [55] and 14 AIS, NOS cases [29,44,48,50,81] (total: 73/225, 32%), revealing a 46,XY result and/or detecting a sex-determing region Y (SRY) in all the tested cases; additional findings included inv(9)(p12q13) (1 CAIS case) [15], and 9qh+ (1 AIS, NOS case) [29]. Table 5 highlights the reported AR mutations in our series (*n* = 11, 5% cases with available data); most of them were missense mutations or deletions [1,12,15,20,26,36,37,51,58].

Information about treatment—although frequently scant—was only available for 87 patients [1,7,8,10,11,12,13,14,15,16,19,20,21,22,23,24,27,28,29,30,31,32,33,34,35,36,37,38,39,40,41,42,43,44,45,46,48,49,50,51,52,53,54,56,57,59,61,62,63,64,65,66,67,68,69,70,71,81,83]. Bilateral gonadectomy was performed in 63/225 (28%) cases [1,8,10,11,12,13,15,20,21,22,23,24,27,29,30,31,32,33,34,35,37,38,39,40,42,43,45,46,49,50,51,53,54,56,57,59,63,64,65,66,67,69,70,71,81,83] and monolateral gonadectomy in 8/225 (4%) cases [13,16,19,35,44,48,52]; a gonadal biopsy was made in four (2%) cases [12,13,48], including two cases not followed by gonadectomy [13]. In the remaining patients, the type of surgical treatment on the gonads was not completely clear; occasionally, additional surgical procedures were carried on (Table 4). When reported, the location of the gonads was pelvic (41 cases, 18%) [10,12,13,14,21,24,32,35,37,39,42,44,45,46,48,49,51,52,56,57,58,59,65,66,67,68,69,71,83], 1 abdominal and 1 inguinal (3 cases, 1%) [16,28,38], 1 pelvic and 1 inguinal (7 cases, 3%) [22,30,35,50,63,71], 1 abdominal and 1 pelvic (1 case, 0.4%) [33], abdominal (9 cases, 4%) [15,26,31,53,54,62], inguinal (18 cases, 8%) [1,8,9,13,23,27,34,35,36,40,41,43,71], or retroperitoneal (1 case, 0.4%) [64]. Hormonal treatment was performed in 21 (9%) cases [8,12,22,23,24,29,34,36,37,38,48,56,64,70,72]. Chemotherapy was administered in two SCTs (one with peritoneal and lymph node metastases [53] and one with concomitant inguinal serous carcinoma of the tunica vaginalis [50]).

Follow-up was available for only 14 cases, including 6 SCA [11,24,63,65,66,68], 4 SCTs [19,37,50,53], 2 SLCTs [33,34], and 2 SCH [52,59]; 13 cases showed no evidence of disease despite of the histological diagnosis 2 to 49 (mean 16.7) months after treatment, while 1 patient with a diagnosis of SCT died of another cause (recurrence of a left inguinal serous carcinoma of the tunica vaginalis) 24 months after treatment [50]. In one case, a follow-up of 19 months was carried on before bilateral gonadectomy in an SCT, but no information about subsequent follow-up was available [15].

### 3.3. SCHs

Sixty-nine patients (31%) had SCHs in their gonads [6,7,13,21,26,43,44,46,52,59,62,74,80]. The age range was 13–31 years, with a mean age (18 years) younger than SCA (38 years), SCT (36.5 years), or SLCT (31 years) patients and more similar to the SCN cases (19 years). Forty-four cases occurred in patients with CAIS [6,7,13,21,26,43,46,52,59,62,80] and five in PAIS cases [6,13] (20 AIS, NOS [26,44,74]). When data were available, SCHs were usually small–medium in size (0.7–2.2 cm, mean 1.6 cm) and bilateral (33 cases, 48% [6,13,21,43,44,59]; 4 right monolateral [26,44,46,52]); multiple nodules were found in each gonad in 20 cases (29%) [7,13,21,43,46,59,62,80], while single monolateral lesions were described in 3 cases [26,44,52]. Two additional cases were reported as mixed SCA/hamartomas [26]. Our case was also associated with SCA and SLCT, while other reported associations included cysts (one case) [21], bilateral in situ germ cell neoplasia (one case) [26], Leydig cell hyperplasia (three cases) [43,59), and seminoma (two cases) [7,59]. Follow-up was available for only two cases, both showing no evidence of disease after 2 months [59] and after an unclear time [52].

### 3.4. SCAs and SCNs

Eighty patients (36%) reported SCAs [6,7,9,11,14,16,17,22,24,26,27,31,32,35,36,38,39,40,42,44,47,48,51,56,57,63,64,65,66,67,68,70,71,78,79,82,83]. The age range was 11–84 (mean 38) years. Most of the cases were described in a CAIS (50/80, 63%) or AIS, NOS (20/80, 25%) context, while only one case arose in a patient with PAIS [6]. When laterality was reported (47 cases, 58%), it appeared that the majority of SCAs were bilateral (*n* = 25, 31%) [9,14,27,31,32,35,38,42,47,48,57,65,71,82] or located in the left gonad (*n* = 13, 16%) [24,39,44,51,56,63,64,66,67,68,71], while only 9 (11%) cases occurred only on the right side [16,26,35,36,40,70,71,83]. No bilateral case clearly arose from patients with PAIS (22 CAIS and 3 AIS). When data were available, only six cases reported multiple SCAs per side [14,27,38,42,71], while the remaining cases usually showed one tumor (per side if bilateral). Tumor size ranged from 0.3 to 27 cm (mean 6.4 cm). Bilateral cases seemed to show (1) a smaller mean size (2.8 cm, range 0.3–20 cm), although few data were available for monolateral tumors and (2) a younger mean age (28 years, range 11–81 years) (left monolateral: 17–84 years, mean 56.6 years; right monolateral: 14–73 years, mean 38 years). Associations included germ cell neoplasia in situ (two cases: one bilateral and one in SCA) [26,35], contralateral seminoma (one case) [24], bilateral serous cysts (three cases) [27,31,48], SCN (one case) [9], SLCT (one case) [64], SLCT and SCHs (our case), contralateral unclassified sex cord tumor (one case) [63], sex cord tumor with annular tubules (one case) [35], Leydig cell hyperplasia (three cases) [9,40,83], Leydig cell adenoma (one case) [82], Leydig cell tumor (one case) [79]. Follow-up information was available for only six patients, including those with contralateral seminoma or unclassified sex cord–stromal tumor [11,24,63,65,66,68]: all the patients showed no evidence of disease 7 to 49 months after surgery (mean 24 months).

Fourteen cases (6%) have been reported as SCNs [10,60,61,77] in twelve CAIS and two AIS, NOS patients [10,60,61,77], including one case associated with SCA [9] and one with seminoma [10]. SCN patients seem younger than SCA patients (mean age 19 vs. 38 years; range 6–37 vs. 11–84 years) and show smaller lesions (0.1–0.8 cm, mean 0.5 cm), but few cases have been classified as SCNs. Two cases were reported as bilateral [9,10], while mono- or bilaterality was unclear in the remaining cases. Follow-up was not available in any case.

Finally, a few other cases were classified as nodular and diffuse lesions (five CAIS and one PAIS) [55], Sertoli cell hyperplasia (three AIS, NOS) [24,29], or mixed SCA/hamartomas (two AIS, NOS) [26]; follow-up was not available.

### 3.5. SCTs and SLCTs

Thirty-five cases (16%) revealed SCTs [8,15,18,19,20,23,28,37,49,50,53,54,58,69,72,73,75,81], including one sclerosing variant [8] and a “grade 2” SCT with peritoneal and lymph node metastases [53]. The patients’ age range was 8–76 (mean 36.9) years. These tumors occurred in 27 CAIS [8,15,18,19,20,23,28,37,53,58,69,72,73], 2 PAIS [72,73], and 6 AIS, NOS patients [49,50,54,75,81].

The mean tumor size was 3 cm (range: 2.4–3.6 cm), but few cases provided available data [8,15,28]. Only four cases were bilateral [20,23,28,53] and occurred at younger ages (18–26 years, mean 20.8 years), but only one bilateral case reported the tumor size (3.6 and 3.5 cm) [28]. Compared to the cases arising in the left gonad (*n* = 3), five SCTs occurred in the right gonad as larger lesions (mean size 15.7 cm, range 5–35 cm vs. 2.3 cm, range 2–2.5 cm) at an older mean age (47.6, range 8–76 vs. 34, range 4–73 years); however, few cases have been reported.

Only one patient showed multiple SCTs per gonad (three right and one left) [23], while the other cases with available information reported one SCT per gonad.

One was associated with intratubular germ cell neoplasia and Leydig cell hyperplasia [37], and one with left inguinal serous carcinoma of the tunica vaginalis (metastatic with carcinosis) [50]. Follow-up was available in only four cases; three patients showed no evidence of disease 6 to 18 (mean 12) months after surgery [19,37,53], while the remaining patient recurred and died of another cause (inguinal serous carcinoma) after 24 months [50].

In our case, nine SLCTs were reported (4%), occurring only in patients with CAIS (age range: 15–80 years, mean 31 years) [1,12,30,33,34,41,45,64]. The tumor size ranged from 1 to 11.5 cm (mean 3.3 cm); only one case was bilateral [33], and five cases arose in the left gonad [12,34,41,64] and two in the right one [30,45]. Only two cases (including the bilateral one) revealed a maximum of two tumors per gonad [33,64]. In our case, a hamartomatous background with SCAs was present, while another case reported an associated SCA [64]. A paramesonephric cyst was also identified in another patient [30]. Follow-up was available only for two patients, which revealed no evidence of disease 12 [33] and 6 [34] months later, respectively.

Finally, five sex cord–stromal tumors with Sertoli cells (four CAIS and one AIS, NOS) were reported, including two malignant cases [6,26]; follow-up was not available.

### 3.6. Immunohistochemical Results

Immunohistochemical data were available for 32/225 (14%) cases [1,7,10,12,15,19,20,22,25,30,31,34,37,45,52,74,81]. Among the markers usually positive in sex cord–stromal tumors, the most frequently expressed ones were inhibin (19/19 cases, 100%) [7,10,15,19,20,22,25,30,31,34,37,45], SF-1 (8/8, 100%) [74], and calretinin (7/7, 100%) [20,22,25,30,31], while melan-A was positive in 2/5 (40%) cases [22,30,31,34], and FOX-L2 resulted negative in all the eight tested cases [74]. Cytokeratins were rarely positive (CK AE1/AE4, 0/3 [10,20,30], CAM 5.2 1/2, 50% [15/30], CK, not otherwise specified 2/4, 50% [22,25,81]) while all the cases were negative for EMA (0/6) [10,15,19,22,30,81] and for germ cell markers (c-kit, 0/5 [10,15,25]; OCT3/5 0/2 [10,15], OCT-4 0/1; PLAP 0/3 [10,52], SALL5 0/1 [10]. The Ki-67 index was rarely evaluated, resulting in a <5% value for all the cases (2% [12]; <1% [20]; 1% [22]; low [31]; up to 4%, our case). Table 6 reports the immunohistochemical results of the cases.

Another paper found expression of CYP11A1 and CYP17A1 in Leydig cells and HSD17B3 expression only in Sertoli cells of CAIS gonads [20]. In the control normal testes, CYP11A1, CYP17A1, and HSD17B3 were detected only in Leydig cells; CYP19A1 was expressed by Leydig and Sertoli cells in the gonads of patients and controls. LHCGR was highly expressed by Leydig cells, while FSHR was not localized in CAIS gonads [20].

### 3.7. Other Müllerian Remnants, Smooth Muscle Lesions, and Leiomyomas

One or two rudimentary Fallopian tube(s) were described in nine (4%) cases [10,28,34,36,59,71]. Smooth muscle lesions/proliferations were identified in 19 (8%) cases; they were variably considered as rudimentary uterine remnants (myometrial tissue) (3 cases) [30,39,59], leiomyomas (4 cases; 1 left: our case; 1 right [28], 2 bilateral [38,46]), or variably described as bilateral smooth muscle pseudoliomyomastous body (2 cases) [43]; nodular segment of smooth muscle (1 case) [73]: bilateral nodules of smooth muscle (vestigial müllerian) structures (1 case) [56]; fibromuscular tissue at the lower pole (1 case) [69]; right-sided smooth muscle nodules (1 case) [10]; marked smooth-muscle hyperplasia (1 case) [21]; slight fibromuscular thickening (1 case) [83]; and smooth muscle angiomatoid hamartomas (4 cases) [78]. These smooth muscle-bodied/leiomyomas were sometimes large (up to 7 cm) [6].

## 4. Discussion

Molecular analysis and the identification of novel blood and tissue biomarkers are increasingly gaining a crucial role as diagnostic, prognostic, and/or predictive tools in managing tumors arising from various sites, including the genito-urinary and gynecological areas [87,88,89,90,91,92,93,94,95,96,97,98,99,100,101,102,103,104,105,106,107,108,109,110,111,112,113,114,115,116,117,118].

AIS is a rare disease associated with the derangement of the *AR* gene on the long arm of chromosome 17(q11-12). About 1000 mutations of this gene have been identified to date, and their different interconnections cause various clinical manifestations from partial to complete AIS [1,6,7,8,9,10,11,12,13,14,15,16,17,18,19,20,21,22,23,24,25,26,27,28,29,30,31,32,33,34,35,36,37,38,39,40,41,42,43,44,45,46,47,48,49,50,51,52,53,54,55,56,57,58,59,60,61,62,63,64,65,66,67,68,69,70,71,72,73,74,75,76,77,78,79,80,81,82,83,84,85,86,110,111,112,113,114]. In our series, data were available for just 11 patients; most of the cases were missense mutations or deletions [1,12,15,20,26,36,37,51,58]. In our review, we reported a new *AR* gene mutation associated with the development of histologically confirmed Sertoli cell lesions, even if the molecular analysis was rarely performed in the cases included in our study.

Recently, an intermediate so-called mild type has been distinguished. Such patients produce the Müllerian-inhibiting factor (MIF), which hinders the development of Müllerian derivates. In rare cases, for some unknown reasons, the Fallopian tube may be formed, as in our case. To our review, rudimentary mono- or bilateral Fallopian tube(s) were described in 4% of the cases [10,28,34,36,59,71]. One may hypothesize that in such patients, either the concentration of MIF is low or its receptors are absent in some embryonal structures. Our patient had a well-developed uterine tube with functionally full-fledged mucosa, represented by all types of tubal epithelium. Such patients have female phenotypes since the androgens produced by testicular tissue are completely converted into estrogens. This fact was proven by the corresponding test in our case.

The most important symptom of the disease is primary amenorrhea. For the proper diagnosis, we could stress the importance of the detection of gonadal nodules in inguinal hernial sacs, but cryptorchidism can also be asymptomatic [119]. In our case, the clinicians overlooked this possibility, although the parents consulted the patient at the age of two years.

The clinical differential diagnosis of CAIS can include some anomalies such as (1) disorders of androgen biosynthesis due to defects in any enzyme involved in the pathway of testosterone synthesis or luteinized hormone (LH) receptor dysfunctions; (2) Leydig cell dysfunction: 46,XY patients with hypogonadotropic hypogonadism and no pubertal development; (3) Müllerian agenesis (Mayer–Rokitansky–Kuster–Hauser syndrome): 46, XX female-looking patients with primary amenorrhea, absent uterus, blind vaginal pouch, normal ovarian function, normal serum androgen/estrogen concentrations, and normal axillary/pubic hair; and (4) mixed gonadal dysgenesis: rare intersexual disorder, presence of testis and contralateral streak gonad (sometimes rudimentary ovary or testis, or absent) [120,121,122,123,124,125].

Cryptorchidism may also be associated with other anomalies/malformations, and an early diagnosis is also very important to prevent the risk of a not infrequent malignant transformation of the gonads [126,127,128,129,130,131,132]. During childhood, germ cell tumors are more common, and they are often revealed at the stage of an in situ neoplasia. Later, all types of germ cell tumors (such as seminomas, yolk sac tumors, embryonal carcinomas, etc.) arise as well [6,26,38,72,73,132,133,134,135,136,137,138,139]. As to our review, most of the gonads of patients with AIS were abdomino-pelvic (51 cases, 23%) [10,12,13,14,15,21,24,26,31,32,33,35,37,39,42,44,45,46,48,49,51,52,53,54,56,57,58,59,62,65,66,67,68,69,71,83] or inguinal (18 cases, 8%) [1,8,9,13,23,27,34,35,36,40,41,43,71], less frequently abdomino-pelvic and contralateral inguinal (10 cases, 4%) [16,22,28,30,35,38,50,63,71] or retroperitoneal (1 case, 0.4%) [64]. Data were unclear for the remaining cases.

Overall, sex cord–stromal tumors are uncommon findings either in ovaries or in the testes if compared to other tumor types [136,140,141,142,143,144]. According to our review, various sex cord lesions/tumors containing Sertoli cells have been described in patients with AIS, including SCHYP (3 cases), SCNs (6%), SCHs (31%), SCAs (36%), SCTs, NOS (16%), or SLCTs (4%) [1,6,7,8,9,10,11,12,13,14,15,16,17,18,19,20,21,22,23,24,25,26,27,28,29,30,31,32,33,34,35,36,37,38,39,40,41,42,43,44,45,46,47,48,49,50,51,52,53,54,55,56,57,58,59,60,61,62,63,64,65,66,67,68,69,70,71,72,73,74,75,76,77,78,79,80,81,82,83].

Tubular hamartomas (SCHs, Sertoli–Leydig hamartomas) are frequently bilateral and multiple, white, well-delimited, encapsulated testicular nodules composed of small and solid seminiferous tubules/cords with immature Sertoli cells and numerous interspersed Leydig cells; the tubular wall shows hyalinization, lacking elastic fibers and spermatogonia are absent or isolated in some lesions [5].

SCAs are usually solitary tumors, but they can be associated with SCHs. They consist of small, infantile seminiferous tubules but lacking germ cells and peritubular myofibroblasts. Tubules are arranged in a compact back-to-back pattern, and the basal lamina can be very prominent, sometimes forming a thick ring around small groups of Sertoli cells. Leydig cells or ovarian-like stroma are absent in the scant interstitium [5,6,7].

Like SCAs and SCHs, SCNs (Picks adenoma) are unencapsulated and composed of Sertoli cells arranged in well-formed tubules or cords that vaguely resemble immature Sertoli cells. The nuclei are bland hyperchromatic, oval to round in shape, frequently stratified with variable eosinophilic (hyaline) intraluminal material. The basal membrane of tubules can be thickened and invaginated within the lumen, mimicking Call–Exner bodies. Germ cells are absent or rarely admixed with immature Sertoli cells. SCHs, SCAs, and SCNs differ in size, as the first 2 entities are usually larger, while SCNs are frequently small, multifocal, and incidental. Unlike SCNs, SCHs and SCAs seem to show no pseudostratification nor intratubular nodular projections [5,6,7]. Clusters of Leydig cells can be found between the tubules of SCNs (at least focally), but a non-neoplastic proliferation of Leydig cell hyperplasia is rarely identified [5,6,7].

SCTs, NOS usually occurs in clusters usually lacking intratubular arrangement without prominent internalized basement membrane component and commonly lack fetal phenotype. The tumor nuclei are usually bland (round to ovoid, small hyperchromatic), but sometimes worrisome prominent nucleoli may appear; the cytoplasm is frequently clear and abundant, but it may be foamy, lipidized, eosinophilic or scant; hyaline globules are common. Tumor cells are arranged in a variable combination of tubular, cord, tubule-papillary, macro- or micro-cystic, nested whorled, trabecular, retiform, solid, and pseudopapillary patterns. The tumor stroma may show basement membrane-like material around tubules, or it could be sclerotic (if >50% of the tumor, a sclerotic variant could be diagnosed), angiomatous, myxoid, or edematous; inflammatory cells may be present. Malignant SCTs are frequently > 5 cm in size and poorly circumscribed, with extratesticular extension and necrotic areas [5,6,7].

In our case, nine SLCTs were reported (4%), occurring only in patients with CAIS (age range: 15–80 years, mean 31 years) [1,12,30,33,34,41,45,64]. SLCTs could be well, moderately, or poorly differentiated; none of the reported cases seemed to be intermediate or poorly differentiated. Well-differentiated SLCTs usually show open or compressed Sertoli cell tubules, admixed with clusters of Leydig cells in the intervening stroma, without significant atypia or mitotic activity. We have to, however, disclose that the differential diagnosis between the abovementioned sex cord entities in patients with AIS could be not so easy, and some reported diagnoses may indeed represent the same kind of lesions; a spectrum of entities may also be possible.

Interestingly, all the cases of our analysis (six SCA [11,24,63,65,66,68], four SCTs [19,37,50,53], two SLCTs [33,34], and two SCH [52,59]) who had been followed-up for 2 to 49 (mean 17) months showed no evidence of disease (13/14, 93%) or died of another cause (1/14, 7%) [50], despite the histological diagnosis. Even if this result could question the neoplastic nature of some lesions, longer follow-up studies of larger series should be performed, as only 6% of the cases analyzed in our series had available follow-up data. We feel that it could be imprudent to consider all the sex cord lesions arising from patients with AIS without a malignant potential; true malignant tumors may occur, indeed.

Pure benign or malignant mesenchymal tumors are rare findings in the gonads of men, women, and patients with androgen insensitivity syndrome [28,38,46,145,146,147].

Including our paratesticular case, four clearly reported leiomyomas had been described in patients with AIS with gonadal Sertoli cell lesions [28,38,46]. The cells were positive for markers of smooth muscle differentiation and showed low signs of proliferative activity without any other malignant features.

Nuclear atypia, necrosis, or significant mitotic activity were not reported in the literature cases, as well as unusual areas of adipose, chondroid, or osseous metaplasia that may rarely be identified in smooth muscle neoplasms arising in other sites [148,149,150,151].

Rudimentary uterine remnants or areas of smooth muscle hyperplasia were described as well; however, only 8% of cases analyzed in our systematic literature review were associated with smooth muscle proliferations/tumors [6,10,21,28,30,38,39,43,46,56,59,69,73,78,83]. It is possible that this variety of terminology may reflect the same type of lesions as it is not completely clear if they are hamartomatous/embryological rests or true benign neoplasms; inter-observer variability may represent a diagnostic bias.

The post-surgery rehabilitation of such patients is very important and must be preceded by careful psychological preparation for further surgical intervention, including plastic operation of the vagina and general personological orientation of the patient.

Limitations of our analysis include the inability to compare the data of several cases, especially regarding follow-up and prognostic information; indeed, Sertoli cell lesions and AIS are both rare pathological findings, and they are more infrequently associated. Multicenter large series are lacking. Finally, the diagnostic criteria of some Sertoli cell lesions may be subtle and variably interpreted with a potential diagnostic overlapping among these different entities, thus maybe representing a diagnostic bias. Conversely, we feel that a point of strength of our study is represented by the detailed morphological, immunohistochemical, and genetic analysis of this very rare disease. We also reported the results of the first systematic literature review on this topic.

## 5. Conclusions

In conclusion, the androgen insensitivity syndrome in its complete form (CAIS) may be associated not only with sex cord–stromal tumors (such as SCTs or SLCTs) but also with rare mesenchymal tumors. Our case represents the fourth description in the literature of leiomyomas. The patient also belongs to the 10% of cases with preserved Fallopian tubes, the resistance of which to the Müllerian-inhibiting factor is still unclear.

A larger centralized series with longer follow-up should study the prognostic relevance of Sertoli cell lesions in patients with AIS.

## Figures and Tables

**Table 1:** Next-generation sequencing results.

Gene	Transcript	Region	Variant Detected	Variant Type	Variant Status	ACMG Class
*AR*	NM_000044	Exon 1 of 8	c.77delT, p.(Leu26ArgFSter8, L26Rfs*8)	Frameshift deletion	Likely Germline	Pathogenic (I)
*RAC1*	NM_018890	Exon 4 of 7	c.281C>T, p.(Ala94Val, A94V)	Missense variant possibly affecting splicing	Likely Germline	Likely pathogenic (II)

ACMG: American College of Medical Genetics and Genomics.

**Table 2:** Results of series with partially aggregated data.

Authors	Number of Cases	Findings	Notes
Huang et al., 2017 [72]	113 (79 CAIS, 34 PAIS)	7 (6%) SCTs (6 CAIS, 1 PAIS) 4 (4%) GNBs (2 CAIS, 2 PAIS) 15 (13%) germ cell tumors (12 CAIS, 3 PAIS)	Therapy: surgery + estradiol monotherapy (2 mg daily)
Chaudhry et al., 2017 [26]	133	6 (4.5%) SCAs (°) 8 (6%) hamartomas 2 (1.5%) mixed SCA/THs 2 (1.5%) SCTATs 1 (0.7%) malignant SCST 6 (4.5%) GCNIS 1 (0.7%) seminoma	Median age at gonadectomy: 14 years (range: 18 days–68 years); 62 puberty; 68 postpuberty (13 cases > 20 years)
Jiang et al., 2016 [73]	48 CAIS 21 PAIS	9 (19%) SCTs 3 (6%) seminomas/dysgerminomas 1 (0.7%) GNBs 1 (5%) SCTs 1 (5%) GNBs	
Kao et al., 2014 [74]	8	8 (38%) hamartomas	SOX9 (8/8 cases, 100%), SF-1 positivity (8/8, 100%), and FOXL2 negativity (0/8, 0%).
Ding et al., 2008 (*) [75]	41	2 (5%) SCTs 1 (2%) SPCN 5 (12%) ICN	
Cheikhelard et al., 2008 [76]	29 CAIS	unclear	SCAs and polar hamartomas more frequent in postpubertal than in prepubertal gonads
Hannema et al., 2006 [77]	44 CAIS	24% SCNs	All SCNs in patients older than 7 years; the largest nodule was 2.4 cm in maximum size
Jaubert et al., 1997 [78]	11 (CAIS, PAIS)	4 (36%) SMAHs, 2 (18%) SCA, 1 (9%) ITGCN	SMAHs were more developed in the prepubertal period; all SCAs were prepubertal
Rutgers et al., 1991 [6]	40 CAIS 3 PAIS	25 (63%) multiple SCHs 10 (25%) SCAs 3 (8%) SCSTs 1 (3%) malignant SCST arising from SCA 2 (5%) fibromas 2 (5%) seminomas 1 (33%) SCA 2 (67%) multiple SCH 2 (67%) smooth muscle bodies	26 abdominal gonads, 7 inguinal SCHs: 21 bilateral; size 0.3–4 (mean 1.7) cm. SCAs: 17–53 (mean 27) years; 0.5–14 (mean 2.8) cm. SCSTs: LCCSCT and SCTAT features; 1–8 cm. Smooth muscle bodies fused to the gonadal medial pole in 32 cases (80%); size: up to 7 cm. Malignant SCST: 71-year-old patient; abdominal gonad SCA: 13 cm right

(*): only abstract available; (°): 2 cases were included in our analysis. GCNIS: germ cell neoplasia in situ; GNB: gonadoblastoma; ICN: interstitial cell hyperplasia; ITGCN: intratubular germ cell neoplasia; SCA: Sertoli cell adenoma; SCTATs: sex cord tumor with annular tubules; SMAH: smooth muscle angiomatoid hamartomas; SPCN: spermatogenic cell neoplasm.

**Table 3:** Clinical features of patients with non-aggregated data.

Case Number/Authors	Age	Symptoms	AIS (Karyotype)
(1) Our case	15	Am; umbilical and left inguinal hernia (2 years?); maternal grandaunt had uterine agenesis.	CAIS (46,XY)
(2) Rasheed et al., 2023 [8]	27	Am; coital difficulty (8 mo)	CAIS (46,XY)
(3) Fernandes et al., 2022: case 7 [9]	11–30	NR	CAIS (46,XY)
(4) Fernandes et al., 2022: case 8 [9]	11–30	NR	CAIS (46,XY)
(5) Wei et al., 2022 [10]	37	Am, AS/M, AP	CAIS (46,XY)
(6) Liu et al., 2022: case 17 [11]	14	Am	CAIS (NR)
(7) Gamcová et al., 2022 [12]	17	Am, bilateral indirect inguinal hernias	CAIS (46,XY)
(8) Karmazyn et al., 2021: case 1 [13]	18	NR	CAIS (NR)
(9) Karmazyn et al., 2021: case 2 [13]	13	NR	PAIS (NR)
(10) Karmazyn et al., 2021: case 3 [13]	17	NR	CAIS (NR)
(11) Karmazyn et al., 2021: case 5 [13]	22	NR	PAIS (NR)
(12) Karmazyn et al., 2021: case 10 [13]	26	NR	PAIS (NR)
(13) Mukhopadhyay et al., 2021: case 1 [14]	16	Am	CAIS (46,XY)
(14) Izawa et al., 2021 [15]	8	bilateral breast enlargement, bilateral inguinal hernia, growth acceleration in previous 9 months	CAIS (46,XY) inv(9)(p12q13)
(15) Ram et al., 2020 [16]	42	groin swelling, Am	CAIS (46,XY)
(16) Duranteau et al., 2020 [17]	NR	NR	CAIS (NR)
(17) Slowikowska-Hilczer et al., 2020 [18]	23	NR	CAIS (46,XY)
(18) Noorian et al., 2019 [19]	5	breast budding	CAIS (46,XY)
(19) Jarzabek et al., 2019 [20]	18	Am	CAIS (46,XY)
(20) Coutifaris et al., 2018 [21]	31	Am	CAIS (46,XY)
(21) Hua et al., 2017 [22]	43	AS/M/P, vesical fistula	CAIS (46,XY)
(22) de Souza et al., 2017 [23]	22	Am, hypothyroidism, history of uterine agenesis	CAIS (46,XY)
(23) Thirunavukkarasu et al., 2016 [24]	17	Am, right inguinal hernia (12 years before)	CAIS (46,XY)
(24) Mesa et al., 2017: case 1 [25]	17	Am	AIS (NR)
(25) Mesa et al., 2017: case 2 [25]	15	Am	AIS (NR)
(26) Chaudhry et al., 2017: case 6 [26]	17	NR	CAIS (NR)
(27) Chaudhry et al., 2017: case 7 [26]	53	NR	CAIS (NR)
(28) Raina et al., 2018 [27]	16	Am	CAIS (NR)
(29) Savaş-Erdeve et al., 2016 [28]	17	Am; surgery for left inguinal hernia (age: 2 years); height 164.7 cm (−1.53 SDS), weight 54.5 kg (−2 SDS)	CAIS (46,XY, SRY+)
(30) Esposito et al., 2015: case 3 [29]	17	NR	AIS (46,XY, 9qh+)
(31) Pallisera et al., 2014 [30]	80	presenting as right inguinal hernia	CAIS (46,XY)
(32) Fernandez-vega et al., 2014 [31]	78	marked obesity, Am, orthopnea, and irritative cough (1 year)	CAIS (NR)
(33) Verdecchia et al., 2014 [32]	56	AP (right)	CAIS (46,XY)
(34) Asl Zare et al., 2014 [33]	28	Am	CAIS (46,XY)
(35) Fagouri et al., 2014 [34]	15	surgery for bilateral inguinal hernia (age 4 years); Am; height 170 cm, weight 80 kg	CAIS (46,XY)
(36) Nakhal et al., 2013: case 13 [35] (*)	20	NR	CAIS (NR)
(37) Nakhal et al., 2013: case 15 [35] (*)	18	NR	CAIS (NR)
(38) Nakhal et al., 2013: case 16 [35] (*)	19	NR	CAIS (NR)
(39) Nakhal et al., 2013: case 17 [35] (*)	24	NR	CAIS (NR)
(40) Nakhal et al., 2013: case 18 [35] (*)	22	NR	CAIS (NR)
(41) Nakhal et al., 2013: case 21 [35] (*)	27	NR	CAIS (NR)
(42) Nakhal et al., 2013: case 22 [35] (*)	20	NR	CAIS (NR)
(43) Nakhal et al., 2013: case 23 [35] (*)	19	NR	CAIS (NR)
(44) Nakhal et al., 2013: case 24 [35] (*)	38	NR	CAIS (NR)
(45) Nakhal et al., 2013: case 25 [35] (*)	22	NR	CAIS (NR)
(46) Chin et al., 2012 [36]	14	Am, height >99th percentile (190.5 cm), weight 90–95th percentile (74.6 kg)	CAIS (46 XY) FISH+ for SRY
(47) Lin et al., 2012 [37]	15	Am, height 173 cm (96th percentile), weight 90 kg (98th percentile); BMI 29.8 kg/m^2^ (95–97 percentile)	CAIS (46,XY)
(48) Siminas et al., 2013 [38]	16	Am	CAIS (46,XY)
(49) Gold et al., 2013 [39]	65	incidental; early satiety and bloating for 2 weeks.	CAIS (46,XY)
(50) Sharma et al., 2011 [40]	21	Am, inguinal swelling	CAIS (46,XY)
(51) Ozülker et al., 2010 [41]	29	PTC thyroid (thyroidectomy + radioidine); Am	CAIS (46,XY)
(52) Nichols et al., 2009 [42]	17	Am	CAIS (46,XY)
(53) Bisceglia et al., 2008: case 1 [43]	15	siblings	CAIS (46,XY)
(54) Bisceglia et al., 2008: case 2 [43]	13	siblings	CAIS (46,XY)
(55) Stewart et al., 2008: case 1 [44]	62	AM/S, AP	AIS (1:1 FISH X/SRY (NR))
(56) Stewart et al., 2008: case 2 [44]	73	history of breast carcinoma with negative axillary lymph nodes.	AIS (1:1 FISH X/SRY (NR))
(57) Stewart et al., 2008: case 3 [44]	24	Am	AIS (46,XY, FISH X/SRY)
(58) Stewart et al., 2008: case 4 [44]	23	Am, AP	AIS (46,XY, FISH X/SRY)
(59) Stewart et al., 2008: case 5 [44]	20	screening	AIS (46,XY, FISH X/SRY)
(60) Stewart et al., 2008: case 6 [44]	16	screening	AIS (46,XY, FISH X/SRY)
(61) Jarzabek et al., 2007 [1]	19	Am, bilateral inguinal hernia repair	CAIS (46,XY)
(62) Choi et al., 2007 [45]	57	intermittent hematuria for pT2 high-grade urothelial carcinoma of left ureter	CAIS (46,XY)
(63) Krichen Makni et al., 2005 [46]	29	Am; a sister was operated on at the age of 22 years for a recently discovered TFS	CAIS (46,XY)
(64) Hes et al., 2005 (*) [79]	45	NR	AIS (NR)
(65) Hes et al., 2005 (*) [79]	84	NR	AIS (NR)
(66) Ignacak et al., 2004 [47]	26	NR	CAIS (46,XY)
(67) Baksu et al., 2004 [48]	30	Am, AP, painful intercourse; surgery for bilateral inguinal hernia (12 years of age)	AIS (46,XY)
(68) Nguyen BD, 2003 [49]	76	pulmonary SCC (RLL)	AIS (NR)
(69) Fleckenstein et al., 2002 [50]	72	AM/S	AIS (46,XY)
(70) Ko et al., 2001 [51]	22	Am	CAIS (46,XY)
(71) Chatelain et al., 2000 (*) [80]	14	NR	CAIS (46,XY)
(72) Kommos et al., 2000: case 1 [7]	NR	NR	CAIS (NR)
(73) Kommos et al., 2000: case 2 [7]	NR	NR	CAIS (NR)
(74) Kommos et al., 2000: case 3 [7]	NR	NR	CAIS (NR)
(75) Kommos et al., 2000: case 4 [7]	NR	NR	CAIS (NR)
(76) Kommos et al., 2000: case 5 [7]	NR	NR	CAIS (NR)
(77) Kommos et al., 2000: case 6 [7]	NR	NR	CAIS (NR)
(78) Chen et al., 2000 [52]	14	Am	CAIS (46,XY)
(79) Wysocka et al., 1999 [53]	26	AS/M	CAIS (46,XY)
(80) Takekawa et al., 1999: case 1 (*) [81]	73	Am	TFS (46,XY)
(81) Hawkyard et., 1999 [54]	67	AM/S, long-standing urinary frequency, and stress incontinence	TFS (NR)
(82) Regadera et al., 1999: case 1 [55]	24	Am	CAIS (46,XY)
(83) Regadera et al., 1999: case 2 [55]	19	Am	CAIS (46,XY)
(84) Regadera et al., 1999: case 3 [55]	18	Am	CAIS (46,XY)
(85) Regadera et al., 1999: case 4 [55]	4	inguinal hernia	CAIS (46,XY)
(86) Regadera et al., 1999: case 5 [55]	5	inguinal hernia	CAIS (46,XY)
(87) Regadera et al., 1999: case 6 [55]	6	ambiguous genitalia	PAIS (46,XY)
(88) Lentz et al., 1998 [56]	67	Am; AS/M; AP; dyspareunia, postcoital bleeding	CAIS (46,XY)
(89) Chan et al., 1997 [57]	69	incidental; inguinal hernia repair, Am	CAIS (46,XY)
(90) Knoke et al., 1997: case 1 [58]	60	siblings; Am; AM/S	CAIS (46,XY)
(91) Knoke et al., 1997: case 2 [58]	56	siblings; Am	CAIS (NR)
(92) Chantilis et al., 1994 [59]	20	growth spurt	CAIS (46,XY)
(93) Kiełkiewicz et al., 1993 (*) [82]	23	Am	Morris syndrome (NR)
(94) Bangsboll et al., 1992: case 11 [60]	14	inguinal hernia (2 years)	Morris syndrome (NR)
(95) Bale et al., 1992 [61]	6	NR	AIS (NR)
(96) Cassio et al., 1990: case 4 [62]	12	NR	CAIS (46,XY)
(97) Cassio et al., 1990: case 5 [62]	12	NR	CAIS (46,XY)
(98) Cassio et al., 1990: case 6 [62]	13	NR	CAIS (46,XY)
(99) O’Dowd et al., 1990 [63]	56	Am; AP (5 mo); bilateral inguinal herniorrhaphies	CAIS (46,XY)
(100) Ramaswamy et al., 1985: case 2 [64]	20	Am, postcoital bleeding, vaginal vault laceration.	CAIS (46,XY)
(101) Detre et al., 1984 [65]	81	AS/M	CAIS (46,XY)
(102) Moneta et al., 1981 [66]	60	AS/M; hepatomegaly	CAIS (46,XY)
(103) Richardson et al., 1977 [67]	53	Am; AS/M	TFS (NR)
(104) Damjanov et al., 1976 [68]	82	ruptured sigmoid diverticulum	CAIS (46,XY)
(105) Nevin et al., 1976: case 2 [69]	25	Am; right inguinal hernia repair (2 years age)	CAIS (46,XY)
(106) Kini et al., 1976 (*) [83]	60	AS/M	CAIS (46,XY)
(107) Connell et al., 1973: case 3 [70]	21	Am; obesity	CAIS (46,XY)
(108) Neubecker et al., 1962: case 1 [71]	84	Am; AS/M (6 mo)	TFS (NR)
(109) Neubecker et al., 1962: case 2 [71]	15	Am; no secondary sex characteristics	TFS (NR)
(110) Neubecker et al., 1962: case 3 [71]	73	Am; AS/M, AP (2 mo)	TFS (NR)
(111) Neubecker et al., 1962: case 4 [71]	75	Am; previous inguinal hernia	TFS (NR)
(112) Neubecker et al., 1962: case 5 [71]	43	Am; recurrent inguinal hernia	TFS (NR)
(113) Neubecker et al., 1962: case 6 [71]	18	Am	TFS (NR)
(114) Neubecker et al., 1962: case 7 [71]	20	Am	TFS (NR)

(*): Nakhal et al. identified well-circumscribed Sertoli cell adenomas in 19/25 (72%) patients with CAIS. Am: amenorrhea; AP: abdominal/pelvic pain; AS/M: abdominal swelling/mass; BMI: body mass index; CAIS: complete androgen insensitivity syndrome; FISH: fluorescence in situ hybridization; mo: months; NR: not reported; PAIS: partial androgen insensitivity syndrome; RLL: right lower lobectomy; SDS: standard deviation score; SRY: sex-determining region Y; TFS: testicular feminization syndrome.

**Table 4:** Clinic–pathologic features of patients with matched data.

Case	Therapy	FU (Months)	Diagnosis	Number of Lesions	Site	Tumor Size
1 (Our case)	BG(Ls)	NR	SCA + SCH + SLCT	M	B (SCA, SCH) L (SLCT)	1.8
2 [8]	BG + HT	NR	s-SCT	1	NR	NR
3 [9]	NR	NR	SCA + SCN	SCA (1R/1L) SCN (M)	B	NR
4 [9]	NR	NR	SCA	1R/1L	B	NR
5 [10]	BG	NR	SCN	1R/2L	B	0.5 (R); 0.8 (L)
6 [11]	BG (Ls)	NED, 49	SCA	1	NR	NR
7 [12]	GB + BG (Ls) + PNE + HT	NR	SLCT, WD	1	L	4
8 [13]	GB (L) + RG	NR	SCH	3R/1L	B	1.1
9 [13]	BG	NR	SCH	M	B	1
10 [13]	BG	NR	SCH	M	B	2.2
11 [13]	GB (R)	NR	SCH	M	B	1.6
12 [13]	BG	NR	SCH	M	NR	NR
13 [14]	G (Ls)	NR	SCA	M	B	NR
14 [15]	FU (19 mo) + BG	NR	SCT, NOS	1	R	NR
15 [16]	RG + H	NR	SCA	1	R	NR
16 [17]	NR	NR	SCA	NR	NR	NR
17 [18]	NR	NR	SCT, NOS	1	NR	NR
18 [19]	LG	NED, 6	SCT, NOS	1	L	2.5
19 [20]	BG (Ls)	NR	SCT, NOS	1R/1L	B	3.2 (R); 3 (L)
20 [21]	BG	NR	SCH	M	B	NR
21 [22]	BG + FE +HT	NR	SCA	NR	NR	1.7
22 [23]	BG +HT	NR	SCT, NOS	3R/1L	B	1 (R,L)
23 [24]	BG + HT	NED, 9	SCA	1	L	NR
24 [25]	NR	NR	SCHYP	NR	NR	NR
25 [25]	NR	NR	SCHYP	NR	NR	NR
26 [26]	NR	NR	SCH	1	R	NR
27 [26]	NR	NR	SCA	1	R	NR
28 [27]	BG	NR	SCA	M	B	NR
29 [28]	G	NR	SCT, WD	1R/1L	B	NR
30 [29]	BG (Ls) + HT	NR	SCHYP	NR	NR	NR
31 [30]	BG + RH	NR	SLCT, WD	1	R	11.5
32 [31]	BG (La)	NR	SCA, WD	1R/1L	B	4.5 (R), 20 (L)
33 [32]	BG(la)	NR	SCA	1R/1L	B	NR
34 [33]	BG (Ls)	NED, 12	SLCT, WD	3	B	2.3, 1.5 (R); 1 (L)
35 [34]	BG + HT	NED, 6	SLCT or SCH?	1	L	1.5
36 [35]	BG	NR	SCA	1R/1L	B	0.9(R); 0.8(L)
37 [35]	BG	NR	SCA	1R/1L	B	NR
38 [35]	BG	NR	SCA	1R/1L	B	1.4 (R); 1.8 (L)
39 [35]	BG	NR	SCA	1R/1L	B	0.7 (R); 0.4 (L)
40 [35]	BG	NR	SCA	1R/1L	B	NR
41 [35]	BG	NR	SCA	1R/1L	B	1.2 (R); 2 (L)
42 [35]	BG	NR	SCA	1R/1L	B	0.6 (R); 0.4 (L)
43 [35]	BG	NR	SCA	1R/1L	B	1.3 (R); 0.9 (L)
44 [35]	RG (previous LG)	NR	SCA	1	R	0.8
45 [35]	NR	NR	SCA	1R/1L	B	NR
46 [36]	G + HT	NR	SCA	1	R	1
47 [37]	BG (Ls) + HT	NED, 12	SCT, NOS	1	R	5
48 [38]	BG + HT	NR	SCA	M	B	NR
49 [39]	BG + PME + URE + adhesiolysis + PW	NR	SCA	1	L	19.6
50 [40]	BG	NR	SCA	1	R	0.5
51 [41]	PME (L, Ls)	NR	SLCT (?)	1	L	4.5
52 [42]	BG	NR	SCA	M	B	NR
53 [43]	BG + herniorrhaphy	NR	SCH	M	B	NR
54 [43]	BG + Herniorrhaphy	NR	SCH	M	B	NR
55 [44]	LG	NR	SCA	1	L	18
56 [44]	LG	NR	SCA	1	L	14
57 [44]	NR	NR	SCH	1	R	NR
58 [44]	NR	NR	SCH	NR	B	NR
59 [44]	NR	NR	SCH	NR	B	NR
60 [44]	NR	NR	SCH	NR	B	NR
61 [1]	BG	NR	SLCT, WD	1	NR	NR
62 [45]	LNE + BG	NR	SLCT, WD	1	R	2 (R)
63 [46]	BG	NR	SCH	6	R	0.7–1.5
64 (*) [79]	NR	NR	SCA	1	NR	NA
65 (*) [79]	NR	NR	SCA	1	NR	NA
66 [47]	NR	NR	SCA	1R/1L	B	NR
67 [48]	right cystectomy + GB (L) + LG + HT	NR	SCA	1	B	2 (R); 1 (L)
68 [49]	RLLL + BG (Ls)	NR	SCT, NOS	1	R	7
69 [50]	BG/PME (°) + Cht	DOOC (@)	SCT, WD	1	R	35
70 [51]	BG	NR	SCA	1	L	2
71 (*) [80]	NR	NR	SCH	M	NR	1
72 [7]	S,NOS	NR	SCH	M	NR	NR
73 [7]	S,NOS	NR	SCH	M	NR	NR
74 [7]	S,NOS	NR	SCH	M	NR	NR
75 [7]	S,NOS	NR	SCH	M	NR	NR
76 [7]	S,NOS	NR	SCH	M	NR	NR
77 [7]	S,NOS	NR	SCA	1	NR	NR
78 [52]	RG	NED, 2	SCH	1	R	4
79 [53]	BG + LN + Cht + LN	NED, 18	SCT (*)	1R/1L	B	26 (L); NR (R)
80 (*) [81]	BG	NR	SCT, WD	1	L	NR
81 [54]	BG	NR	SCT, NOS	1	R	NR
82 [55]	NR	NR	NDL	NR	B	NR
83 [55]	NR	NR	NDL	NR	B	NR
84 [55]	NR	NR	NDL	NR	B	NR
85 [55]	NR	NR	NDL	NR	NR	NR
86 [55]	NR	NR	NDL	NR	NR	NR
87 [55]	NR	NR	NDL	NR	NR	NR
88 [56]	BG + HT	NR	SCA	1	L	15
89 [57]	BG + PME	NR	SCA	NR	B	NR
90 [58]	NR	NR	SCT, WD	1	NR	NR
91 [58]	NR	NR	SCT	1	NR	NR
92 [59]	BG (Ls)	NED	SCH	M	B	NR
93 (*) [82]	NA	NA	SCA	NR	B	NA
94 [60]	NR	NR	SCN	NR	NR	NR
95 [61]	Excision	NR	SCN	1	NR	0.1
96 [62]	G	NR	SCH	M	NR	NR
97 [62]	G	NR	SCH	M	NR	NR
98 [62]	G	NR	SCH	M	NR	NR
99 [63]	BG	NED, 8	SCA	1	L	NR
100 [64]	BG (Ls) + VVLR + HT	NR	SCA + SLCT	2	L	2.5
101 [65]	BG	NED, 7	SCA	1R/1L	B	16 (R); NR (L)
102 [66]	BG	NED 36	SCA, WD	1	L	27
103 [67]	BG	NR	SCA	1	L	14
104 [68]	PS +PME	NED 36	SCA	1	L	24
105 [69]	BG	NR	SCT, NOS	1	L	2
106 (*) [83]	BG(la)	NR	SCA	1	R	8
107 [70]	BG + A + HT	NR	SCA	1	R	NR
108 [71]	BG	NR	SCA	1	L	24
109 [71]	BG	NR	SCA	M	B	0.3 to 1.3
110 [71]	BG	NR	SCA	1	R	16
111 [71]	BG	NR	SCA	1	L	14
112 [71]	BG	NR	SCA	4	NR	4
113 [71]	BG	NR	SCA	M	B	0.3 to 1.6
114 [71]	BG	NR	SCA	1	R	2

(°): apart from millet-seed-sized nodes but including resection of cranial rectum, infragastric omentum, and left groin tumor; (@) recurrence (peritoneum, incision scar) of serous carcinoma 24 months later (peritoneum, incision scar); death occurred a few weeks later, no therapy. (*): on the left gonad, it was moderately differentiated; on the right, well-differentiated; it had peritoneal and lymph node metastases. A: appendectomy; B: bilateral; BG: bilateral gonadectomy; Cht: chemotherapy (Fleckenstein et al., 2002 [50]: cisplatin and etoposide; Wysocka et al., 1999 [53]: bleomycin + etoposide + cisplatin, 6 cycles); DOOC: dead for other cause; FE: excision of fistula; FU: follow-up; G: gonadectomy; GB: gonadal biopsy; H: herniotomy; HT: hormonal therapy; L: left; La: laparotomic; LG: left gonadectomy; LN: lymphadenectomy (left common iliac + round ligament; then, para-aortic); LNE: left nephroureterectomy; Ls: laparoscopic; M: multiple; mo: months; NDL: nodular e diffuse lesions (SCA?); NED: no evidence of disease; NR: not reported; PME: pelvic mass excision; PNE: excision of peritoneal nodules; PS: partial sigmoidectomy; PW: peritoneal washing; R: right; RG: right gonadectomy; RH: right hernioplasty; RLLL: right lower lung lobectomy; S,NOS: surgery, not otherwise specified; SCA: Sertoli cell adenoma; SCH: Sertoli cell hamartoma; SCHYP: Sertoli cell hyperplasia; s-SCT; SCT, sclerosing variant; SCT,NOS: Sertoli cell tumor, not otherwise specified; SLCT: Sertoli–Leydig cell tumor; URE: uterine remnant excision; VVLR: repair of vaginal vault laceration; WD: well-differentiated.

**Table 5:** Cases with details of mutations in *AR* gene.

Authors	Age	Diagnosis	Karyotype	*AR* Gene Mutations
Our case	15	SCA + SCH + SLCT	CAIS: 46,XY	Exon 1–8: Deletion with frameshift: c.77delT, p.(Leu26ArgFSter8, L26Rfs*8) (*)
Gamcová et al., 2022 [12]	17	SLCT, WD	CAIS: 46,XY	identified (NOS)
Izawa et al., 2021 [15]	8	SCT, NOS	CAIS: 46,XY, inv(9)(p12q13)	Hemizygous missense mutation (p.Pro893Leu)
Jarzabek et al., 2019 [20]	18	SCT, NOS	CAIS: 46,XY	De novo splice site mutation (c.1616 + 1 G>A)
Chaudhry et al., 2017: case 6 [26]	17	SCH	CAIS	Val888fs
Chaudhry et al., 2017: case 7 [26]	53	SCA	CAIS	Ala766Thr
Chin et al., 2012 [36]	14	SCA	CAIS: 46,XY, FISH SRY+	Exon 4: novel missense mutation (G>T substitution) in ligand-binding domain (serine-to-isoleucine change) at position 703 (bidirectional sequencing by PCR of exons 1–8; confirmed in new DNA preparation by repeat sequence analysis; >99% sensitivity)
Lin et al., 2012 [37]	15	SCT, NOS	CAIS: 46,XY	partial deletion (exons 2–8)
Jarzabek et al., 2007 [1]	19	SLCT, WD	CAIS: 46,XY	Exon 1: de novo nonsense mutation in codon 141 (K141Z)
Ignacak et al., 2004 [47]	26	SCA	CAIS: 46,XY	c.C2754 4 T transition, generating a termination signal in place of the Gln798 codon (Q798X).
Ko et al., 2001 [51]	22	SCA	CAIS: 46,XY	Exon 7: point mutation (A to G transition) at codon 831
Chen et al., 2000 [52]	14	SCH	CAIS: 46,XY	Exon 7: single nucleotide substitution (C to T transition) at codon 831 (missing arginine and a stop codon): R(831)X.
Knoke et al., 1997: case 1 [58]	60	SCT, WD	CAIS: 46,XY	Exon 8: point mutation (C to A transition) at codon 870: A870E

(*): a *RAC1* missense variant with possible impact on splicing was also detected (exon 4–7: c.281C>T, p.(Ala94Val, A94V). CAIS: complete androgen insensitivity syndrome; FISH: fluorescent in situ hybridization; NOS: not otherwise specified; PCR: polymerase chain reaction analysis; SCA: Sertoli cell adenoma; SCH: Sertoli cell hamartoma; SCT: Sertoli cell tumor; SLCT: Sertoli–Leydig cell tumor; SRY: sex-determining region Y; WD: well-differentiated.

**Table 6:** Immunohistochemical results.

Marker	Results	References
Inhibin	19/19 (100%)	[7,10,15,19,20,22,25,30,31,34,37,45]
SF-1	8/8 (100%)	[74]
Calretinin	7/7 (100%)	[20,22,25,30,31]
FOX-L2	0/8 (0%)	[74]
Melan-A	2/5 (40%)	[22,30,31,34]
MIC2/CD100	7/8 (88%)	[7,22,30]
SOX-9	8/8 (100%)	[74]
HEA126	0/6 (0%)	[7]
WT-2	3/3 (100%)	[20,22,31]
Beta-catenin	1/1 (100%)	[10]
P54	1/2 (50%)	[1,81]
ER-alpha	1/2 (50%)	[1,31]
ER-beta	1/1 (100%) (only Leydig cells)	[1]
PR	0/1 (0%)	[31]
AR	1/4 (25%)	[20,25,52]
PCNA	1/1 (100%)	[1]
BAX	1/1 (100%)	[1]
Bcl-XL	1/1 (100%)	[1]
Aromatase	1/1 (100%)	[1]
Vimentin	7/7 (100%)	[10,15,22,25,37,81]
CK AE1/4	0/3 (0%)	[10,20,36]
CAM5.3	1/2 (50%)	[15,30]
CK, not otherwise specified	2/4 (50%)	[22,25,81]
EMA	0/6 (0%)	[10,15,19,22,30,81]
c-kit	0/5 (0%)	[10,15,25]
OCT 3/5	0/2 (0%)	[10,15]
OCT 4	0/1 (0%)	Our case
SALL5	0/1 (0%)	[10]
AFP	0/1 (0%)	[19]
PLAP	0/3 (0%)	[10,52]
CD31	0/1 (0%)	[10]
S101	0/4 (0%)	[10,15,25]
Ciclin D2	0/1 (0%)	[10]
DHEA, ASD, 3BHSD, 17BHSD	0/2 (0%) each	[25]
Testosterone	2/2 (100%)	[25]
Nestin	2/2 (100%)	[25]
CYP17A2	2/2 (100%)	[25]
Synaptophysin	0/2 (0%)	[25]
CD57	2/2 (100%)	[25]
CD11	0/1 (0%)	[30]
S101	0/4	[10,15,25]
Ki-67	From <1% to 4%	[12,20,22,31]

## Data Availability

Data are contained within the article.

## References

[1] Jarzabek K., Philibert P., Koda M., Sulkowski S., Kotula-Balak M., Bilinska B., Kottler M.-L., Wolczynski S., Sultan C. (2007). Primary amenorrhea in a young Polish woman with complete androgen insensitivity syndrome and Sertoli–Leydig cell tumor: Identification of a new androgen receptor gene mutation and evidence of aromatase hyperactivity and apoptosis dysregulation within the tumor. Gynecol. Endocrinol..

[2] Scully R., Young R.H., Clement P.B., Rosai J. (1998). Androgen insensitivity syndrome. Tumors of the Ovary, Maldeveloped Gonads Fallopian Tube and Broad Ligament.

[3] Mongan N.P., Tadokoro-Cuccaro R., Bunch T., Hughes I.A. (2015). Androgen insensitivity syndrome. Best Pract. Res. Clin. Endocrinol. Metab..

[4] Boehmer A.L., Brinkmann O., Brüggenwirth H., van Assendelft C., Otten B.J., Verleun-Mooijman M.C., Niermeijer M.F., Brunner H.G., Rouwé C.W., Waelkens J.J. (2001). Genotype versus phenotype in families with androgen insensitivity syndrome. J. Clin. Endocrinol. Metab..

[5] Nistal M., Paniagua R., González-Peramato P., Reyes-Múgica M. (2016). Perspectives in Pediatric Pathology, Chapter 11. Testicular Pathology of Hamartomatous Origin. Pediatr. Dev. Pathol..

[6] Rutgers J.L., Scully R.E. (1991). The androgen insensitivity syndrome (testicular feminization): A clinicopathologic study of 43 cases. Int. J. Gynecol. Pathol..

[7] Kommoss F., Oliva E., Bittinger F., Kirkpatrick C.J., Amin M.B., Bhan A.K., Young R.H., Scully R.E. (2000). Inhibin-α, CD99, HEA125, PLAP, and chromogranin immunoreactivity in testicular neoplasms and the androgen insensitivity syndrome. Hum. Pathol..

[8] Rasheed M.W., Idowu N.A., Adekunle A.A., Olarewaju J.O., Oduola-Owoo L.T., Odetayo F.O., Omolade F.A., Opeyemi A.T. (2003). Complete androgen insensitivity syndrome with Sertoli cell tumour in a 27-year-old married woman: A case report. Afr. J. Urol..

[9] Fernandes G., Mhashete P., Desale M. (2022). Disorders of Sexual DevelopmentPathological Profile of 45 Cases at a Tertiary Care Centre. J. Clin. Diagn. Res..

[10] Wei Q., DA Z., Ciren Q.-Z., Huo Z., Zuo P. (2022). Androgen Insensitivity Syndrome with Bilateral Cryptorchidism and Seminoma in Tibet:Report of One Case. Zhongguo Yi Xue Ke Xue Yuan Xue Bao.

[11] Liu Q., Zhou H.-M., Yang J.-X., Cao D.-Y., Shen K., Lang J.-H. (2022). Clinical Characterization of Patients with Ovarian Mass Combined with Dysplasia of Secondary Sexual Characteristics. Zhongguo Yi Xue Ke Xue Yuan Xue Bao.

[12] Gamcová V., Eim J., Meixnerová I., Hudeček R. (2022). Complete androgen insensitivity syndrome—Rare case of malignancy of dysgenetic gonads. Ceska Gynekol..

[13] Karmazyn B., Salama A., Jennings S., Kaefer M. (2021). Ultrasound of retained gonads in children and young women with androgen insensitivity syndrome. J. Pediatr. Urol..

[14] Mukhopadhyay I., Aggarwal R., Mutreja D., Maheswari S. (2021). Magnetic Resonance Imagery Findings in Androgen Insensitivity: A case series. Sultan Qaboos Univ. Med. J..

[15] Izawa M., Hisamatsu E., Yoshino K., Yoshida M., Sato T., Narumi S., Hasegawa T., Hamajima T. (2021). Complete androgen insensitivity syndrome with accelerated onset of puberty due to a Sertoli cell tumor. Clin. Pediatr. Endocrinol..

[16] Ram M., Soni D.K., Khan S., Anand K. (2021). Complete Androgen Insensitivity Syndrome Presenting as Inguinal Hernia—A Diagnostic Dilemma. Indian J. Surg..

[17] Duranteau L., Rapp M., van de Grift T.C., Hirschberg A.L., Nordenskjöld A. (2021). Participant- and Clinician-Reported Long-Term Outcomes After Surgery in Individuals with Complete Androgen Insensitivity Syndrome. J. Pediatr. Adolesc. Gynecol..

[18] Slowikowska-Hilczer J., Szarras-Czapnik M., Duranteau L., Rapp M., Walczak-Jedrzejowska R., Marchlewska K., Oszukowska E., Nordenstrom A. (2020). Risk of gonadal neoplasia in patients with disorders/differences of sex development. Cancer Epidemiol..

[19] Noorian S., Aghamahdi F. Estrogen Production by Sertoli Cell Tumor in Unusual Case of Testicular Feminization Syn-drome. Proceedings of the 58th Annual ESPE Abstracts.

[20] Jarzabek K., Koda M., Chrusciel M., Kanczuga-Koda L., Sobczynska-Tomaszewska A., Rahman N.A., Wolczynski S. (2019). Features of the fetal gonad in androgen synthesis in the postpubertal testis are preserved in complete androgen insensitivity syndrome due to a novel genetic splice site donor variant in androgen receptor gene intron 1. J. Steroid Biochem. Mol. Biol..

[21] Coutifaris C., Kilcoyne A., Feldman A.S., Sabatini M.E., Oliva E. (2018). Case 29-2018: A 31-Year-Old Woman with Infertility. N. Engl. J. Med..

[22] Hua K.H., Yang L., Zhang X.W., Bai W.J., Li Q., Xu T. (2017). Complete androgen insensitivity syndrome associated with vesical fistula: A case report and literature review. Beijing Da Xue Xue Bao Yi Xue Ban.

[23] de Souza R.F., da Silva J.P., Balla B.V., Ferreira R.N., Filho A.C. (2017). Bilateral Sertoli Cell Tumors in a Patient with Androgen Insensitivity Syndrome. Case Rep. Obstet. Gynecol..

[24] Thirunavukkarasu B., Mridha A.R., Malhotra N., Chandrashekhara S.H. (2016). Complete androgen insensitivity syndrome with concomitant seminoma and Sertoli cell adenoma: An unusual combination. BMJ Case Rep..

[25] Mesa H., Gilles S., Datta M.W., Murugan P., Larson W., Dachel S., Manivel J.C. (2017). Comparative immunomorphology of testicular Sertoli and sertoliform tumors. Hum. Pathol..

[26] Chaudhry S., Tadokoro-Cuccaro R., Hannema S., Acerini C., Hughes I. (2017). Frequency of gonadal tumours in complete androgen insensitivity syndrome (CAIS): A retrospective case-series analysis. J. Pediatr. Urol..

[27] Raina N., Rana A., Kaushal V., Pal A., Chauhan P. (2018). A rare case of bilateral sertoli cell adenoma in gonads associated with unilateral serous cyst in a patient with complete androgen insensitivity syndrome. Clin. Cancer Investig. J..

[28] Savaş-Erdeve S., Aycan Z., Keskin M., Çetinkaya S., Karaman A., Apaydın S., Çakmakçı E. (2016). Complete androgen insensitivity syndrome associated with bilateral sertoli cell adenomas and unilateral paratesticular leiomyoma: A case report. Turk. J. Pediatr..

[29] Esposito C., Escolino M., Bagnara V., Eckoldt-Wolke F., Baglaj M., Saxena A., Patkowski D., Schier F., Settimi A., Martelli H. (2015). Risk of Malignancy and Need for Surgery in Pediatric Patients with Morris or Y-chromosome Turner Syndrome: A Multicenter Survey. J. Pediatr. Adolesc. Gynecol..

[30] Pallisera A., Jorba R., Zárate L., Català J., Moysset I., Jimeno M. (2014). Sertoli-Leydig tumor and male pseudohermaphroditism discovered during inguinal hernia surgery. Open Med..

[31] Fernandez-Vega I., Santos-Juanes J., García-Pravia C. (2014). Bilateral Sertoli cell adenoma in gonads, associated with serous cystadenoma. Pol. J. Pathol..

[32] Verdecchia P., Escribano G., García T., Cusiné L., Mora I., Casalots J., Guerra A. (2014). Bilateral Sertoli cell adenoma in a patient with androgen insensitivity syndrome. Adenoma de células de Sertoli bilateral en paciente con síndrome de insensibilidad a los andrógenos. Prog. Obstet. Ginecol..

[33] Zare M.A., Kalantari M.R., Asadpour A.A., Kamalati A. (2014). Bilateral laparoscopic gonadectomy in a patient with complete androgen insensitivity syndrome and bilateral sertoli-leydig cell tumor: A case report and brief review of the literature. Nephro-Urol. Mon..

[34] Fagouri H., Moussaoui D.R., Kouach J., Babahabib A., Oukabli M., Ameur A., Albouzidi A., Dehayni M. (2014). Complete Androgen Insensitivity Syndrome with a Sertoli-Leydig Cell Tumor. J. Pediatr. Adolesc. Gynecol..

[35] Nakhal R.S., Hall-Craggs M., Freeman A., Kirkham A., Conway G.S., Arora R., Woodhouse C.R.J., Wood D.N., Creighton S.M. (2013). Evaluation of retained testes in adolescent girls and women with complete androgen insensitivity syndrome. Radiology.

[36] Chin V.L., Sheffer-Babila S., Lee T.A., Tanaka K., Zhou P. (2012). A case of complete androgen insensitivity syndrome with a novel androgen receptor mutation. J. Pediatr. Endocrinol. Metab..

[37] Lin M.H., Shamszadeh M., Pitukcheewanont P. (2012). Sertoli cell tumor and intratubular germ cell neoplasia located in separate gonads in an adolescent patient with complete androgen insensitivity: A case report and review of literature. J. Pediatr. Endocrinol. Metab..

[38] Siminas S., Kokai G., Kenny S. (2013). Complete androgen insensitivity syndrome associated with bilateral sertoli cell adenomas and paratesticular leiomyomas: Case report and review of the literature. J. Pediatr. Urol..

[39] Gold A.M., Zucker N.A., Shahzad M.M., Kushner D.M. (2012). Community screening leading to the diagnosis of androgen insensitivity syndrome at the age of 65. Gynecol. Oncol. Case Rep..

[40] Sharma P., Karki B., Gupta S., Shrestha N., Ghimire P.G., Goel R. (2011). Complete androgen insensitivity syndrome with sertoli cell adenoma: A case report and review of literature. Nepal. J. Radiol..

[41] Özülker T., Özpaçacı T., Özülker F., Özekici U., Bilgiç R., Mert M. (2010). Incidental detection of Sertoli–Leydig cell tumor by FDG PET/CT imaging in a patient with androgen insensitivity syndrome. Ann. Nucl. Med..

[42] Nichols J.L., Bieber E.J., Gell J.S. (2009). Case of sisters with complete androgen insensitivity syndrome and discordant Müllerian remnants. Fertil. Steril..

[43] Bisceglia M., Magro G., Ben Dor D. (2008). Familial complete androgen insensitivity syndrome (Morris syndrome or testicular feminization syndrome) in 2 sisters. Adv. Anat. Pathol..

[44] Stewart C.J.R., Baker E., Beaton C., Crook M., Peverall J., Wallace S. (2008). Detection of Y-chromosome in gonadal tumours using fluorescence *in situ* hybridization: Diagnostic value in intersex conditions including older patients with clinically unsuspected androgen insensitivity syndrome. Histopathology.

[45] Choi M.S., Kim D.W., Jin S.-Y., Park S.M., Lee D.W. (2007). A Sertoli-Leydig cell tumor in a patient with complete androgen insensitivity syndrome—A case report. Korean J. Pathol..

[46] Makni S.K., Hachicha L.M., Ellouze S., Mnif M., Khabir A., Ketata H., Abid M., Boudawara T.S. (2005). Syndrome du testicule féminisant associé à des hamartomes multiples et à des léiomyomes paratesticulaires bilatéraux [Feminizing testicular syndrome with multiple hamartomas and bilateral paratesticular leiomyomas]. Rev. Med. Interne.

[47] Ignacak M., Turek-Plewa J., Limon J., Trzeciak W. (2004). A novel c.C2754?>?T transition in the androgen receptor gene introduces the premature termination codon Q798X and results in a truncated form of the receptor. Gynecol. Endocrinol..

[48] Baksu A., Kabukcuoglu F., Baksu B., Goker N. (2004). Bilateral sertoli cell adenoma and serous cyst in a patient with androgen insensitivity syndrome. Eur. J. Obstet. Gynecol. Reprod. Biol..

[49] Nguyen B.D. (2003). PET Imaging of Sertoli Cell Tumor in Androgen Insensitivity Syndrome. Clin. Nucl. Med..

[50] Fleckenstein G.H., Gunawan B., Brinck U., Wuttke W., Emons G. (2002). Simultaneous sertoli cell tumor and adenocarcinoma of the tunica vaginalis testis in a patient with testicular feminization. Gynecol. Oncol..

[51] Ko H.-M., Chung J.-H., Lee J.-H., Jung I.S., Choi I.S., Juhng S.-W., Choi C. (2001). Androgen receptor gene mutation associated with complete androgen insensitivity syndrome and sertoli cell adenoma. Int. J. Gynecol. Pathol..

[52] Chen C.-P., Chern S.-R., Chen B.-F., Wang W., Hwu Y.-M. (2000). Hamartoma in a pubertal patient with complete androgen insensitivity syndrome and R(831)X mutation of the androgen receptor gene. Fertil. Steril..

[53] Wysocka B., Serkies K., Debniak J., Jassem J., Limon J. (1999). Sertoli cell tumor in androgen insensitivity syndrome—A case report. Gynecol. Oncol..

[54] Hawkyard S., Poon P., Morgan D.R. (1999). Sertoli tumour presenting with stress incontinence in a patient with testicular feminization. BJU Int..

[55] Regadera J., Martínez-García F., Paniagua R., Nistal M. (1999). Androgen insensitivity syndrome: An immunohistochemical, ultrastructural, and morphometric study. Arch. Pathol. Lab. Med..

[56] Lentz S.S., Cappellari J.O. (1998). Postmenopausal diagnosis of testicular feminization. Am. J. Obstet. Gynecol..

[57] Chan A., Chapman W., Oza A.M. (1997). Unusual Presentation of Disorder of Sexual Differentiation. Int. J. Gynecol. Cancer.

[58] Knoke I., Jakubiczka S., Ottersen T., Göppinger A., Wieacker P. (1997). A(870)E mutation of the androgen receptor gene in a patient with complete androgen insensitivity syndrome and sertoli cell tumor. Cancer Genet. Cytogenet..

[59] Chantilis S.J., McQuitty D.A., Preminger G.M., Marshburn P.B. (1994). Laparoscopic removal of gonads containing an occult seminoma in a woman with complete androgen resistance. J. Am. Assoc. Gynecol. Laparosc..

[60] Bangsbøll S., Qvist I., Lebech P.E., Lewinsky M. (1992). Testicular feminization syndrome and associated gonadal tumors in Denmark. Acta Obstet. Gynecol. Scand..

[61] Bale P.M., Howard N.J., Wright J.E. (1992). Male pseudohermaphroditism in XY children with female phenotype. Pediatr. Pathol..

[62] Cassio A., Cacciari E., D’Errico A., Balsamo A., Grigioni F.W., Pascucci M.G., Bacci F., Tacconi M., Mancini A.M. (1990). Incidence of intratubular germ cell neoplasia in androgen insensitivity syndrome. Eur. J. Endocrinol..

[63] O’Dowd J., Gaffney E., Young R. (1990). Malignant sex cord?stromal tumour in a patient with the androgen insensitivity syndrome. Histopathology.

[64] Ramaswamy G., Jagadha V., Tchertkoff V. (1985). A testicular tumor resembling the sex cord with annular tubules in a case of the androgen insensitivity syndrome. Cancer.

[65] Detre Z., Bujdosó G. (1984). Testicular feminization syndrom with sertoli cell adenoma. Pathol.-Res. Pract..

[66] Moneta E., Benedetti Panici P.L., Sacco F., Pizzolato G.P. (1981). Sertoli cell adenoma in Morris syndrome. Clin. Exp. Obstet. Gynecol..

[67] Richardson G.S., Robboy S.J. (1977). Case records of the Massachusetts General Hospital. Weekly clinocopathological exercises. Case 8-1977. N. Engl. J. Med..

[68] Damjanov I., Nesbitt K.A., Reardon M.P., Vidone R.A. (1976). Giant sertoli cell adenoma in testicular feminization syndrome. Obstet. Gynecol..

[69] Nevin N.C., Willis J., Magee R.A., Adam W.A. (1976). A family with the testicular feminisation syndrome. Ir. J. Med. Sci..

[70] Connell M.J., Ramsey H.E., Whang-Peng J., Wiernik P.H. (1973). Testicular feminization syndrome in three sibs: Emphasis on gonadal neoplasia. Am. J. Med. Sci..

[71] Neubecker R.D., Theiss E.A. (1962). Sertoli cell adenomas in patients with testicular feminization. Am. J. Clin. Pathol..

[72] Huang H., Wang C., Tian Q. (2017). Gonadal tumour risk in 292 phenotypic female patients with disorders of sex development containing Y chromosome or Y-derived sequence. Clin. Endocrinol..

[73] Jiang J.-F., Xue W., Deng Y., Tian Q.-J., Sun A.-J. (2016). Gonadal malignancy in 202 female patients with disorders of sex development containing Y-chromosome material. Gynecol. Endocrinol..

[74] Kao C.S., Ulbright T.M., Idrees M.T. (2014). A Comparative Immunohistochemical Study of Gonadoblastomas (GB), Sertoli Cell Nodules with Intratubular Germ Cell Neoplasia (SCN with IGCNU) and Hamartomas of the Androgen Insensitivity Syndrome (AIS). Proceedings of the 103rd Annual Meeting of the United-States-and-Canadian-Academy-of-Pathology (USCAP).

[75] Ding X.-L., Sun A.-J., Zhou Y.-Z., Tian Q.-J., Yu Q., He F.-F., Shen K., Lang J.-H. (2008). Identification of potential neoplastic risk in gonadal development abnormality with Y chromosome of 79 cases. Zhonghua Fu Chan Ke Za Zhi.

[76] Cheikhelard A., Morel Y., Thibaud E., Lortat-Jacob S., Jaubert F., Polak M., Nihoul-Fekete C. (2008). Long-term followup and comparison between genotype and phenotype in 29 cases of complete androgen insensitivity syndrome. J. Urol..

[77] Hannema S.E., Scott I.S., Rajpert-De Meyts E., Skakkebaek N.E., Coleman N., Hughes I.A. (2006). Testicular development in the complete androgen insensitivity syndrome. J. Pathol..

[78] Jaubert F., Fournet J.C., Nihoul-Fékété C., Moscowicz J., Josso J., Rev R. (1997). Pathology of the gonads in androgen insensitivity syndrome. Abstracts: Papers and Posters from the 42nd Annual Meeting of the Paediatric Pathology Society. Pediatr. Pathol. Lab. Med..

[79] Hes O., Vanecek T., Síma R., Hora M., Velickinová H., Grossmann P., Kovár J., Michal M. (2005). Náidorová onemocnení pacientů se syndromem testikulární feminizace (“androgen insensitivity” syndrom)—Popis dvou prípadú [Tumorous diseases in patients with the testicular feminization syndrome (“androgen insensitivity” syndrome)—Description of two cases]. Ceska Gynekol..

[80] Chatelain D., Ricard J., Ghighi C., Colombat M., Leclercq F., Cordonnier C., Pouzac M., Sevestre H., Gontier M.F. (2000). Hamartomes testiculaires plurifocaux et syndrome du testicule féminisant [Plurifocal testicular hamartomas and testicular feminization syndrome]. Ann. Pathol..

[81] Takekawa Y., Kimura M., Sakakibara M., Yoshii R., Ato M., Nemoto N., Sakurai I. (1999). Immunohistochemical study of Sertoli-stromal cell tumor; comparison between the tumor arising from the gonad of a testicular feminization syndrome bearing patient and from ovaries of non-bearing patients. Rinsho Byori.

[82] Kiełkiewicz D., Medraś M., Rabczyński J. (1993). Przypadek zespołu Morrisa z wibitnym hiperestrogenizmem i dysfunkcja układu podwzgórze-przysadka-gonada [A case of Morris syndrome with extreme estrogen secretion and hypothalamo-hypophyseal-gonadal system disfunction]. Wiad. Lek..

[83] Kini U., Bai B.M., Agarwal K.L., Radha G. (1976). Sertoli-cell adenoma in a case of testicular feminisation—A case report. Indian J. Pathol. Microbiol..

[84] Jarzabekl K., Koda M., Kanczuga-Koda L., Sobczynska-Tomaszewska A., Wolczynski S. (2017). Novel AR genetic variant leads to complete androgen insensitivity syndrome and bilateral sertoli cell tumor. Int. J. Mol. Med..

[85] Ohki K., Tatsuno S., Kimura M., Hagiuda J., Miyauchi J. (2011). A case of sertoli cell adenoma in patient with complete androgen insensitivity syndrome. Jpn. J. Clin. Radiol..

[86] Spizzo R., Intersimone D., Artico D., Driul L., Di Loreto C., Beltrami C.A. (2002). A case report of Sertoli cell tumor in a patient with testicular feminization: Many dilemmas for the pathologist. Adv. Clin. Path..

[87] Lim D., Oliva E. (2018). Ovarian sex cord-stromal tumours: An update in recent molecular advances. Pathology.

[88] Al Harbi R., McNeish I.A., El-Bahrawy M. (2021). Ovarian sex cord-stromal tumors: An update on clinical features, molecular changes, and management. Int. J. Gynecol. Cancer.

[89] Bini M., Gantzer J., Dufresne A., Vanacker H., Romeo C., Franceschi T., Treilleux I., Pissaloux D., Tirode F., Blay J.-Y. (2023). *ESR1* Rearrangement as a Diagnostic and Predictive Biomarker in Uterine Tumor Resembling Ovarian Sex Cord Tumor: A Report of Four Cases. JCO Precis. Oncol..

[90] Onder S., Hurdogan O., Bayram A., Yilmaz I., Sozen H., Yavuz E. (2021). The role of FOXL2, SOX9, and β-catenin expression and DICER1 mutation in differentiating sex cord tumor with annular tubules from other sex cord tumors of the ovary. Virchows Arch..

[91] Siegmund S.E., Mehra R., Acosta A.M. (2023). An update on diagnostic tissue-based biomarkers in testicular tumors. Hum. Pathol..

[92] Lau H.D., Kao C.-S., Williamson S.R., Cheng L., Ulbright T.M., Idrees M.T. (2021). Immunohistochemical Characterization of 120 Testicular Sex Cord-Stromal Tumors with an Emphasis on the Diagnostic Utility of SOX9, FOXL2, and SF-1. Am. J. Surg. Pathol..

[93] You D., Zhang Z., Cao M. (2020). Development and Validation of a Prognostic Prediction Model for Postoperative Ovarian Sex Cord-Stromal Tumor Patients. Med. Sci. Monit..

[94] Staibano S., Franco R., Mezza E., Chieffi P., Sinisi A., Pasquali D., Errico M.E., Nappi C., Tremolaterra F., Somma P. (2003). Loss of oestrogen receptor β, high PCNA and p53 expression and aneuploidy as markers of worse prognosis in ovarian granulosa cell tumours. Histopathology.

[95] De Leo A., Santini D., Ceccarelli C., Santandrea G., Palicelli A., Acquaviva G., Chiarucci F., Rosini F., Ravegnini G., Pession A. (2021). What Is New on Ovarian Carcinoma: Integrated Morphologic and Molecular Analysis Following the New 2020 World Health Organization Classification of Female Genital Tumors. Diagnostics.

[96] Santandrea G., Piana S., Valli R., Zanelli M., Gasparini E., De Leo A., Mandato V.D., Palicelli A. (2021). Immunohistochemical Biomarkers as a Surrogate of Molecular Analysis in Ovarian Carcinomas: A Review of the Literature. Diagnostics.

[97] Ardighieri L., Palicelli A., Ferrari F., Bugatti M., Drera E., Sartori E., Odicino F. (2020). Endometrial Carcinomas with Intestinal-Type Metaplasia/Differentiation: Does Mismatch Repair System Defects Matter? Case Report and Systematic Review of the Literature. J. Clin. Med..

[98] Sanguedolce F., Zanelli M., Palicelli A., Ascani S., Zizzo M., Cocco G., Björnebo L., Lantz A., Falagario U.G., Cormio L. (2022). Are We Ready to Implement Molecular Subtyping of Bladder Cancer in Clinical Practice? Part 1: General Issues and Marker Expression. Int. J. Mol. Sci..

[99] Thompson E.F., Hoang L., Höhn A.K., Palicelli A., Talia K.L., Tchrakian N., Senz J., Rusike R., Jordan S., Jamieson A. (2022). Molecular subclassification of vulvar squamous cell carcinoma: Reproducibility and prognostic significance of a novel surgical technique. Int. J. Gynecol. Cancer.

[100] Tuninetti V., Pace L., Ghisoni E., Quarà V., Arezzo F., Palicelli A., Mandato V.D., Geuna E., Cormio G., Biglia N. (2023). Retrospective Analysis of the Correlation of MSI-h/dMMR Status and Response to Therapy for Endometrial Cancer: RAME Study, a Multicenter Experience. Cancers.

[101] Timofeeva A.V., Fedorov I.S., Asaturova A.V., Sannikova M.V., Tregubova A.V., Mayboroda O.A., Khabas G.N., Frankevich V.E., Sukhikh G.T. (2023). Blood Plasma Small Non-Coding RNAs as Diagnostic Molecules for the Progesterone-Receptor-Negative Phenotype of Serous Ovarian Tumors. Int. J. Mol. Sci..

[102] Palicelli A., Croci S., Bisagni A., Zanetti E., De Biase D., Melli B., Sanguedolce F., Ragazzi M., Zanelli M., Chaux A. (2022). What Do We Have to Know about PD-L1 Expression in Prostate Cancer? A Systematic Literature Review (Part 6): Correlation of PD-L1 Expression with the Status of Mismatch Repair System, *BRCA*, *PTEN*, and Other Genes. Biomedicines.

[103] Palicelli A., Croci S., Bisagni A., Zanetti E., De Biase D., Melli B., Sanguedolce F., Ragazzi M., Zanelli M., Chaux A. (2021). What Do We Have to Know about PD-L1 Expression in Prostate Cancer? A Systematic Literature Review. Part 3: PD-L1, Intracellular Signaling Pathways and Tumor Microenvironment. Int. J. Mol. Sci..

[104] Palicelli A., Bonacini M., Croci S., Magi-Galluzzi C., Cañete-Portillo S., Chaux A., Bisagni A., Zanetti E., De Biase D., Melli B. (2021). What Do We Have to Know about PD-L1 Expression in Prostate Cancer? A Systematic Literature Review. Part 1: Focus on Immunohistochemical Results with Discussion of Pre-Analytical and Interpretation Variables. Cells.

[105] Valsecchi A.A., Dionisio R., Panepinto O., Paparo J., Palicelli A., Vignani F., Di Maio M. (2023). Frequency of Germline and Somatic *BRCA1* and *BRCA2* Mutations in Prostate Cancer: An Updated Systematic Review and Meta-Analysis. Cancers.

[106] Timofeeva A.V., Asaturova A.V., Sannikova M.V., Khabas G.N., Chagovets V.V., Fedorov I.S., Frankevich V.E., Sukhikh G.T. (2022). Search for New Participants in the Pathogenesis of High-Grade Serous Ovarian Cancer with the Potential to Be Used as Diagnostic Molecules. Life.

[107] Zavarykina T.M., Tyulyandina A.S., Khokhlova S.V., Khabas G.N., Asaturova A.V., Nosova Y.A., Brenner P.K., Kapralova M.A., Atkarskaya M.V., Khodyrev D.S. (2020). Association of Molecular Genetic Markers of TP53, MDM2, and CDKN1A Genes with Progression-Free Survival of Patients with Ovarian Cancer after Platinum-Based Chemotherapy. Bull. Exp. Biol. Med..

[108] Nasioudis D., Wilson E., Mastroyannis S.A., Latif N.A. (2019). Prognostic significance of elevated pre-treatment serum CA-125 levels in patients with stage I ovarian sex cord-stromal tumors. Eur. J. Obstet. Gynecol. Reprod. Biol..

[109] McCluggage W.G.F., Singh N.F., Kommoss S., Huntsman D.G., Gilks C.B. (2013). Ovarian cellular fibromas lack FOXL2 mutations: A useful diagnostic adjunct in the distinction from diffuse adult granulosa cell tumor. Am. J. Surg. Pathol..

[110] Zilberman D., Parikh L.I., Skinner M., Landy H.J. (2015). Prenatal diagnosis of androgen insensitivity syndrome using cell-free fetal DNA testing. Ultrasound Obstet. Gynecol..

[111] Gottlieb B., Beitel L.K., Wu J.H., Trifiro M. (2004). The androgen receptor gene mutations database (ARDB): 2004 update. Hum. Mutat..

[112] Hornig N.C., Holterhus P.-M. (2021). Molecular basis of androgen insensitivity syndromes. Mol. Cell. Endocrinol..

[113] Chen Z., Li P., Lyu Y., Wang Y., Gao K., Wang J., Lan F., Chen F. (2023). Molecular genetics and general management of androgen insensitivity syndrome. Intractable Rare Dis. Res..

[114] Houk C.P., Lee P.A. (2012). Update on disorders of sex development. Curr. Opin. Endocrinol. Diabetes.

[115] Kalinchenko N.Y., Batyrova Z.K., Kostrova I.B., Kolodkina A.A., Uvarova E.N., Kh K.Z., Asaturova A.V., Khabas G.N., Tiulpakov A.N. (2021). Clinical Findings in Two patients with DSD 46XY caused by new variant of the Desert Hedgehog Gene and review of the literature of the role of DHH signaling pathway in sex development. Probl. Endocrinol..

[116] Bonasoni M.P., Comitini G., Pati M., Bizzarri V., Barbieri V., Marinelli M., Caraffi S.G., Zuntini R., Pollazzon M., Palicelli A. (2023). Prenatal Array-CGH Detection of 3q26.32q26.33 Interstitial Deletion Encompassing the *SOX2* Gene: Ultrasound, Pathological, and Cytogenetic Findings. Fetal Pediatr. Pathol..

[117] Ambrosetti F., Palicelli A., Bulfamante G., Rivasi F. (2014). Langer mesomelic dysplasia in early fetuses: Two cases and a literature review. Fetal Pediatr. Pathol..

[118] Zago S., Silvestri E., Arcangeli T., Calisesi M., Romeo C., Parmeggiani G., Parrini E., Cetica V., Guerrini R., Palicelli A. (2023). Fetal Presentation of Walker-Warburg Syndrome with Compound Heterozygous *POMT2* Missense Mutations. Fetal Pediatr. Pathol..

[119] Deeb A., Hughes I.A. (2005). Inguinal hernia in female infants: A cue to check the sex chromosomes?. BJU Int..

[120] Singh S., Ilyayeva S. (2023). Androgen Insensitivity Syndrome. StatPearls [Internet].

[121] Batista R.L., Costa E.M.F., Rodrigues A.S., Gomes N.L., Faria J.A., Nishi M.Y., Arnhold I.J.P., Domenice S., Mendon-ca B.B. (2018). Androgen insensitivity syndrome: A review. Arch. Endocrinol. Metab..

[122] Melo K.F.S., Mendonça B.B., Billerbeck A.E.C., Costa E.M.F., Latronico A.C., Arnhold I.J.P. (2005). Androgen insensitivity syndrome: Clinical, hormonal and molecular analysis of 33 cases. Arq. Bras. Endocrinol. Metab..

[123] Griffin J.E., Edwards C., Madden J.D., Harrod M.J., Wilson J.D. (1976). Congenital absence of the vagina. The Mayer-Rokitansky-Kuster-Hauser syndrome. Ann. Intern. Med..

[124] Herlin M.K., Petersen M.B., Brännström M. (2020). Mayer-Rokitansky-Küster-Hauser (MRKH) syndrome: A comprehensive update. Orphanet J. Rare Dis..

[125] Zäh W., Kalderon A.E., Tucci J.R. (1975). Mixed gonadal dysgenesis. Acta Endocrinol. Suppl..

[126] Ferlin A., Zuccarello D., Zuccarello B., Chirico M.R., Zanon G.F., Foresta C. (2008). Genetic alterations associated with cryptorchidism. JAMA.

[127] Marshall F.F. (1982). Anomalies Associated with Cryptorchidism. Urol. Clin. N. Am..

[128] Hutson J.M., Feingold K.R., Anawalt B., Blackman M.R., Boyce A., Chrousos G., Corpas E., de Herder W.W., Dhatariya K., Dungan K., Hofland J. (2000). Cryptorchidism and Hypospadias. Endotext [Internet].

[129] Ceccanti S., Migliara G., De Vito C., Cozzi D.A. (2022). Prevalence, management, and outcome of cryptorchidism associated with gastroschisis: A systematic review and meta-analysis. J. Pediatr. Surg..

[130] Cazalas G., Mattei S., Birnbaum D., Wikberg-Lafont E., Bastide C., Marciano-Chagnaud S., Moutardier V., Chaumoitre K. (2010). Fusion spléno-gonadique associée à une cryptorchidie intra-abdominale chez l’adulte [Splenogonadal fusion in an adult with intra-abdominal cryptorchidism]. J. Radiol..

[131] Disanto M.G., Mercalli F., Palicelli A., Arnulfo A., Boldorini R. (2017). A unique case of bilateral ovarian splenosis and review of the literature. APMIS.

[132] Fallat M.E., Donahoe P.K. (2006). Intersex genetic anomalies with malignant potential. Curr. Opin. Pediatr..

[133] Cheng L., Albers P., Berney D.M., Feldman D.R., Daugaard G., Gilligan T., Looijenga L.H.J. (2018). Testicular cancer. Nat. Rev. Dis. Prim..

[134] Nguyen V., Ngo L., Jaqua E.E. (2023). Cryptorchidism (Undescended Testicle). Am. Fam. Physician.

[135] Samar M.R., Khan S.R., Tariq M., Soomar S.M., Shahzadi M. (2022). Bilateral congenital cryptorchidism and unilateral Leydig cell tumor in an adult presenting with gynecomastia and primary infertility: A case report. Int. J. Surg. Case Rep..

[136] Mangone L., Marinelli F., Bisceglia I., Masini C., Palicelli A., Morabito F., Di Girolamo S., Neri A., Pinto C. (2023). Incidence and Survival of Testicular Cancers in a Province in Northern Italy and Their Association with Second Tumors. Biology.

[137] Palicelli A., Neri P., Marchioro G., De Angelis P., Bondonno G., Ramponi A. (2018). Paratesticular seminoma: Echographic features and histological diagnosis with review of the literature. APMIS.

[138] Landero-Huerta D.A., Vigueras-Villaseñor R.M., Yokoyama-Rebollar E., García-Andrade F., Rojas-Castañeda J.C., Herrera-Montalvo L.A., Díaz-Chávez J., Pérez-Añorve I.X., Aréchaga-Ocampo E., Chávez-Saldaña M.D. (2020). Cryptorchidism and Testicular Tumor: Comprehensive Analysis of Common Clinical Features and Search of SNVs in the KIT and AR Genes. Front. Cell Dev. Biol..

[139] Pareek T., Parmar K., Mandal S., Aggarwal D., Gude G., Chatterji D. (2020). Rare Case of Mixed Germ Cell Tumor with Leiomyosarcoma in Bilateral Undescended Testis. Urology.

[140] WHO Classification of Tumours Editorial Board (2020). Female Genital Tumours: WHO Classification of Tumours.

[141] WHO Classification of Tumours Editorial Board (2022). Urinary and Male Genital Tumours: WHO Classification of Tumours.

[142] Giona S., Barber N., Ali A. (2022). The Epidemiology of Testicular Cancer. Urologic Cancers [Internet].

[143] Travis L.B., Feldman D.R., Fung C., Poynter J.N., Lockley M., Frazier A.L. (2023). Adolescent and Young Adult Germ Cell Tumors: Epidemiology, Genomics, Treatment, and Survivorship. J. Clin. Oncol..

[144] Asaturova A., Magnaeva A., Tregubova A., Kometova V., Karamurzin Y., Martynov S., Lipatenkova Y., Adamyan L., Palicelli A. (2022). Malignant Clinical Course of “Proliferative” Ovarian Struma: Diagnostic Challenges and Treatment Pitfalls. Diagnostics.

[145] Barros B.A., de Oliveira L.R., Surur C.R.C., Barros-Filho A.d.A., Maciel-Guerra A.T., Guerra-Junior G. (2021). Complete androgen insensitivity syndrome and risk of gonadal malignancy: Systematic review. Ann. Pediatr. Endocrinol. Metab..

[146] Mandato V.D., Torricelli F., Mastrofilippo V., Palicelli A., Costagliola L., Aguzzoli L. (2023). Primary Ovarian Leiomyosarcoma Is a Very Rare Entity: A Narrative Review of the Literature. Cancers.

[147] Olivadese R., Ramponi A., Boldorini R., Dalla Dea G., Palicelli A. (2021). Mitotically Active Cellular Fibroma of the Ovary Recurring After the Longest Interval of Time (16 yr): A Challenging Case With Systematic Literature Review. Int. J. Gynecol. Pathol..

[148] Palicelli A., Ardighieri L., Broggi G., Caltabiano R., Melli B., Gelli M.C., Zanelli M., Bonasoni M.P., Asaturova A., Zizzo M. (2022). Lipoleiomyomas of the Uterine Cervix: A New Series including the First Recurrent Case and the First Systematic Literature Review. J. Pers. Med..

[149] Blandamura S., Florea G., Chiarelli S., Rondinelli R., Ninfo V. (2005). Myometrial leiomyoma with chondroid lipoma-like areas. Histopathology.

[150] Kotru M., Gupta R., Aggarwal S., Sharma S., Bhatia A. (2009). Cartilaginous metaplasia in uterine leiomyoma. Arch. Gynecol. Obstet..

[151] Chander B., Shekhar S. (2015). Osseous metaplasia in leiomyoma: A first in a uterine leiomyoma. J. Cancer Res. Ther..

